# Beyond here and now: evaluating pollution estimation across space and time from street view images with deep learning

**DOI:** 10.1016/j.scitotenv.2023.166168

**Published:** 2023-08-14

**Authors:** Ricky Nathvani, D Vishwanath, Sierra N. Clark, Abosede S. Alli, Emily Muller, Henri Caste, James E Bennett, James Nimo, Josephine Bedford Moses, Solomon Baah, Allison Hughes, Esra Suel, Antje Barbara Metzler, Theo Rashid, Michael Brauer, Jill Baumgartner, George Owusu, Samuel Agyei-Mensah, Raphael E. Arku, Majid Ezzati

**Affiliations:** 1 Department of Epidemiology and Biostatistics, School of Public Health, Imperial College London, London, UK; 2 MRC Centre for Environment and Health, School of Public Health, Imperial College London, London, UK; 3 Department of Environmental Health Sciences, School of Public Health and Health Sciences, University of Massachusetts, Amherst, USA; 4 Department of Physics, University of Ghana, Accra, Ghana; 5 Centre for Advanced Spatial Analysis, University College London, London, UK; 6 School of Population and Public Health, University of British Columbia, Vancouver, Canada; 7 Institute for Health and Social Policy, McGill University, Montreal, Canada; 8 Department of Epidemiology, Biostatistics, and Occupational Health, McGill University, Montreal, Canada; 9 Institute of Statistical, Social & Economic Research, University of Ghana, Accra, Ghana; 10 Department of Geography and Resource Development, University of Ghana, Accra, Ghana; 11 Regional Institute for Population Studies, University of Ghana, Accra, Ghana

**Keywords:** Deep learning, computer vision, air pollution, noise pollution, street-view images, environmental modelling

## Abstract

Advances in computer vision, driven by deep learning, allows for the inference of environmental pollution and its potential sources from images. The spatial and temporal generalisability of image-based pollution models is crucial in their real-world application, but is currently understudied, particularly in low-income countries where infrastructure for measuring the complex patterns of pollution is limited and modelling may therefore provide the most utility. We employed convolutional neural networks (CNNs) for two complementary classification models, in both an end-to-end approach and as an interpretable feature extractor (object detection), to estimate spatially and temporally resolved fine particulate matter (PM_2.5_) and noise levels in Accra, Ghana. Data used for training the models were from a unique dataset of over 1.6 million images collected over 15 months at 145 representative locations across the city, paired with air and noise measurements. Both end-to-end CNN and object-based approaches surpassed null model benchmarks for predicting PM_2.5_ and noise at single locations, but performance deteriorated when applied to other locations. Model accuracy diminished when tested on images from locations unseen during training, but improved by sampling a greater number of locations during model training, even if the total quantity of data was reduced. The end-to-end models used characteristics of images associated with atmospheric visibility for predicting PM_2.5_, and specific objects such as vehicles and people for noise. The results demonstrate the potential and challenges of image-based, spatiotemporal air pollution and noise estimation, and that robust, environmental modelling with images requires integration with traditional sensor networks.

## 1 Introduction

The urban population in low- and middle-income countries (LMICs) increased from 357 million in 1950 to 3.39 billion in 2020, with the majority of the LMIC population now living in cities (United Nations, Department of Economic and Social Affairs, & Population Division, 2019). While cities offer their inhabitants better access to infrastructure, services and economic opportunity (Ezzati et al., 2018), factors such as road transport and residential and commercial energy generation can also increase hazardous environmental exposures, including air and noise pollution (Kammen and Sunter, 2016; Kelly and Zhu, 2016). Although some sources of urban pollution in LMICs, such as vehicular traffic, are similar to those of many high income countries, there are also differences in the sources, and in their spatial and temporal patterns (Alli et al., 2021; Amegah and Agyei-Mensah, 2017; Clark et al., 2021; Deng et al., 2020; Ebare et al., 2011; Weagle et al., 2018; Zhou et al., 2013) such as seasonal Saharan Desert dust storms (Zhou et al., 2013), burning biomass fuels for cooking and heating, and the use of diesel generators where there are intermittent electricity outages (Dionisio et al., 2010).

Data on the patterns of air and noise pollution and their sources across space and time are needed to identify and evaluate mitigation measures and policies. However, collecting such data is challenging in resource-constrained settings (Brauer et al., 2019; Clark et al., 2020; Khan et al., 2018). Recent methodological advances in image processing and analysis, particularly in the form of deep convolutional neural networks, have demonstrated that street-level images can help with predicting air and noise pollution levels (Ganji et al., 2020; Hong et al., 2020; Qi and Hankey, 2021; Weichenthal et al., 2019), contingent on initial data measurements needed to develop the image-based pollution estimation models. So far, image-based pollution models have largely been developed for East Asia (Chakma et al., 2017; Feng et al., 2021; Gu et al., 2019; Liu et al., 2016, 2015; Wang et al., 2022; Won et al., 2022; Zhang et al., 2018) and North America (Ganji et al., 2020; Hong et al., 2020; Qi and Hankey, 2021), typically based on a few weeks’ observation at selected locations, asynchronous or spatially distant from pollution measurements. Few studies have sought to predict spatially and temporally resolved pollution from images, and none in Africa, the world’s fastest urbanising region (United Nations, Department of Economic and Social Affairs, & Population Division, 2019).

We developed and evaluated machine learning models to predict temporally and spatially varying noise and fine particulate matter (PM_2.5_; particles <2.5 μm in diameter, with known human health impacts (Pope and Dockery, 2006)) levels from street-level images in Accra, Ghana. We used deep convolutional neural networks (CNNs), which learn robust and hierarchical feature representations that give them superior performance for many image-processing tasks (Schmidhuber 2015; Gu et al. 2018), in two complementary strategies. The first used a CNN, without a priori assumptions on the image features relevant for prediction, and another used gradient boosted machines, applied to interpretable image features in the form of object counts, from applying an object-detection CNN to each image. These models were applied to a bespoke dataset of over 1.6 million time-lapsed images co-located with PM_2.5_ and noise measurements, at 145 representative locations over 15 months (Clark et al., 2020). Models were trained and evaluated on subsets of data specifically to interrogate their temporal and spatial generalisability and in order to compare and contrast strategies for data collection with fixed resources when developing such models. We further assessed model performance for both the day and night time, different seasons, and types of urban land use.

## 2 Data and Methodological Context and Contributions

Some studies have predicted pollution from visual elements of the environment. Two studies, also from Accra, recorded PM_2.5_ and PM_10_ in selected neighbourhoods, in a multi-week measurement campaign (Dionisio et al., 2010; Rooney et al., 2012), together with researcher observations and census data on environmental factors, such as biomass fuels and unpaved roads, to predict pollution levels. Some studies have also predicted pollution using remote sensing data, which differs from our study, not only in the view of the city, but also in spatial and temporal scales and the observable features in images (Sorek-Hamer et al., 2022; Wei et al., 2020; Weigand et al., 2019).

Other studies used terrestrial images for predicting air pollution (Chakma et al., 2017; Feng et al., 2021; Ganji et al., 2020; Gu et al., 2019; Hong et al., 2020; Liu et al., 2016, 2015; Qi and Hankey, 2021; Wang et al., 2022; Won et al., 2022; Zhang et al., 2018), and one for noise (Hong et al., 2020) based on images and pollution measurement data though none had spatiotemporally linked image and pollution data during the night time, as we do. Previously adopted approaches span a variety of experimental configurations, making a unifying, quantitative comparison among studies infeasible. The specific metric of pollution (e.g., black carbon vs PM), timescales on which pollution is predicted (single measurement in time vs variation across day), spatial resolution (city-wide vs local), images used (static vs time-varying), data inputs (solely images vs inclusion of meteorological variables), temporal range (<~1 week vs multiple months of observation), synchrony between data sources (pollution and images <~5 min apart vs >~1 year apart), modelling approach (regression of continuous pollution data vs classification into different classes) and model inputs (specific features vs entire images), vary from study to study. Furthermore, within studies that used images as model inputs, a variety of features and feature extraction methods (object detection vs segmentation) were used, including in relation to stationarity of features in time (e.g., buildings and trees vs vehicles and pedestrians). The majority of studies used a single configuration from such choices depending on the available data, generating prediction tasks that are easier or more difficult relative to others. We outline the different experimental setups for previous studies in Appendix Table A. In the specific case of cities in Africa, one study used street-view images to predict PM_2.5_ and NO_2_ across several cities, including Accra (Suel et al., 2022). Data used for model training were derived from modelled estimates of annual average pollution level with a model only evaluated, not trained, on data from Accra.

Our work advances the state of knowledge in a number of ways. Our dataset is much larger and was collected over a longer duration than most previous image-based studies, comprising 145 locations and a total (prior to merging with our pollution data) of 2.1 million images over 15 months. We co-captured both air pollution and noise data with images in both day and night time. We predicted air pollution concentrations and noise levels at finer classification intervals, i.e. with classes that each encompass a smaller and more precise range as described in Section 3.3, than comparable previous classification-based studies. We systematically evaluated both the spatial and temporal generalisability of models which is relevant for designing an optimal digital surveillance strategy and guiding data collection. Our study is unique in the use of both end-to-end CNN (outcome-driven) and object-based (feature-driven) models, which both inform model selection and enhance model interpretability. Finally, to our knowledge, this is the first use of images for predicting both air and noise pollution in the context of an African city.

## 3 Materials and Methods

### 3.1 Data

We collected co-located time-lapsed images at 5-min intervals and PM_2.5_ and noise measurements averaged and recorded at 1-min intervals in a field campaign from April 2019 to June 2020, details of which are described in Appendix A and the study protocol paper (Clark et al., 2020). We had ten fixed sites where data were collected over 15 months, and 135 rotating sites where data was collected for one week. The fixed sites provided information for assessing temporal generalisability of models, and both fixed and rotating sites for assessing spatial generalisability. Sites were grouped into four land-use classes: commercial, business, industrial (CBI); informal, mostly high-density, settlements and slums; formal, mostly low- and mediumdensity, residential areas; and “other” areas that are often peri-urban or rural, and can have dense vegetation (i.e., forest, grassland) or barren land (i.e., sand, soil, dirt). The classes for each fixed site are detailed in Appendix Table B.

### 3.2 Research questions

We developed two types of models that used images to predict noise and air pollution. We analysed how well our models’ prediction generalise across time and space, through the following research questions:

la) Temporal generalisability: How well do models trained on images taken from a single location predict noise and PM_2.5_ at different, random times at the same location?

lb) Spatial generalisability: How well do models trained in 1a), which are based on a single location, generalise to another unseen location?

2a) Spatial generalisability: How well do models trained using an abundance (~1,000,000 total across sites) of images from a set of nine (long-term) fixed sites, predict noise and PM_2.5_ at the remaining (10^th^) unseen location?

2b) Spatial generalisability: How well can models trained using fewer images (~100,000 total across sites) from ~90% of our 135 rotating sites, predict noise and PM_2.5_ at the remaining ~10% of sites?

The fixed sites, due to their extended data collection period, comprised seven times as much data as rotating sites in total. Since in-situ pollution measurements are resource intensive, especially in quantities needed to train a CNN (Sun et al., 2017), there is a need to optimally allocate the use of cameras and pollution measurement hardware, as well as personnel time. Therefore we also investigated whether models trained using more data from a smaller number of (fixed) sites, or fewer data from a greater number of (rotating) sites led to more spatially generalisable CNN models:

2c) Comparison of model types from 2a) and 2b): Do models perform better on multiple, unseen locations (remaining ~10% of rotating sites) when given an abundance of images from a few locations, or fewer images across a variety of locations?

**Figure 1:**
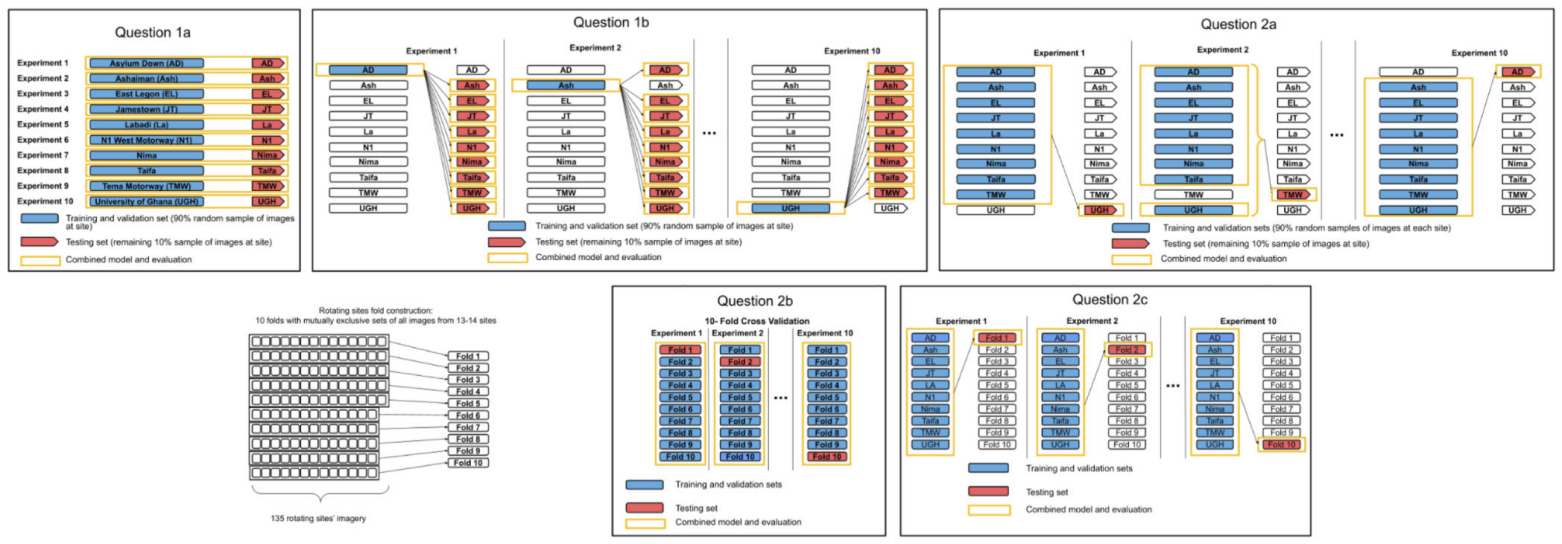
Data use for training and testing of models.

For each question, we divided our data into subsets for training and testing, as illustrated in Figure 1. The number of images belonging to each of the datasets is given in Appendix Table B.

Each panel shows how data from fixed and rotating sites were allocated to training and testing sets, for each question posed in Section 3.2. For the training sets, indicated in blue, each block was further divided into a 75-25 split with the latter being used as a validation set during training configuration and hyperparameter determination. Final models were trained on the entire training set (including the validation set) and evaluated on the testing set, indicated in red.

### 3.3 Modelling

For all research questions, we trained models to predict levels of noise (dBA) or PM_2.5_ (μg/m^3^) at a given time (1-min averaged interval) and location from a single image taken within 30 s of pollution measurement. We framed the prediction task as a classification problem, i.e. the models predict specific ranges (classes) in which noise and PM_2.5_ fall rather than as a continuous value, for two reasons. First, policy targets and guidelines, such as those of the World Health Organization (Basner and McGuire, 2018; World Health Organization, 2021), tend to be formulated based on discrete levels. Second, a preliminary analysis indicated that models trained explicitly for classification outperformed regression models trained for continuous value prediction, as detailed in Appendix C. The classes for noise were: ≤39, 40 to <45, 45 to <50, 50 to <55, 55 to <60, 60 to <65, 65 to <70, 70 to <75, 75 to <80, ≥80 dBA. The classes for PM_2.5_ were: 0 to <5, 5 to <10, 10 to <15, 15 to <20, 20 to <25, 25 to <30, 30 to <40, 40 to <50, 50 to <100, 100 to <150, ≥150 μg/m^3^.

**Figure 2:**
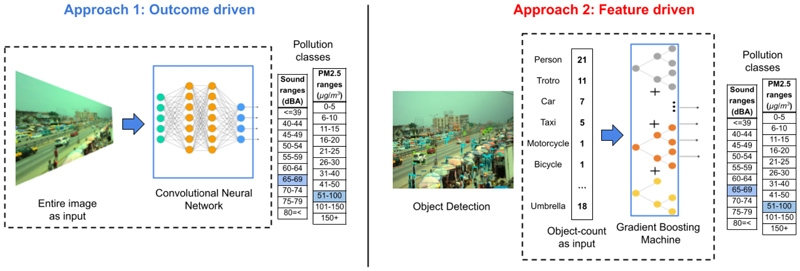
End-to-end (CNN) and object-based (GBM) modelling approaches.

For both forms of pollution, we produced two classification models (Figure 2). The first, referred to as end-to-end classification, used an entire unprocessed image, with red, green and blue pixel channels, as input to a CNN to predict pollution class. No assumptions were made on relevant image features, which were learned from the data. The second group of models used counts of objects detected from images as input for classification via gradient boosted machines (Friedman, 2001) (GBM). Other approaches to feature extraction from images, such as semantic segmentation, could also have been employed to provide model inputs for pollution estimation, as used in a North American study (Qi and Hankey, 2021). We used objects in our second approach since the data needed to train a model, namely objects, were less resource intensive to generate within our bespoke dataset with bounding boxes (Nathvani et al., 2022) as compared with pixel-level annotation, which may also be explored in future work. The object counts were obtained from training an object detection CNN, described in detail in previous work (Nathvani et al., 2022), for object categories relevant to the local environmental context: persons, market vendor (a person carrying a container over their heads which is a common scene in African markets), car, taxi, pick-up truck, bus, lorry, van, tro-tro (mini buses used for public transportation), motorcycle, bicycle, market stall, loudspeaker, umbrella (commonly used to protect market and roadside vendors from the sun and rain), cookstove, cooking pot/bowl (which frequently contain wares for sale in the marketplace), food, trash, (piece of) debris, and animal. All object categories are those which may vary over time at a given place, since although other static features, such as buildings or trees, may also affect noise and air pollution, their unchanging presence over daily timescales is less informative for predicting temporal variation in pollution at a single location (such as those models developed in 1a and 1b). The accuracy with which these objects could be detected in our images is given in Appendix Table C and described in previous work (Nathvani et al., 2022). In this analysis, we did not use counts of cookstove, loudspeakers, market vendors or buses, due to their sparse presence in our data (<10 counts of each in 2.1 million images). The end-to-end and feature-driven approaches are complementary with respect to flexibility and feature agnosticism versus prior assumption and interpretability (Zhang and Zhu, 2018).

### 3.4 Data preparation

We prepared our data in the following manner for the purpose of training and evaluating both CNN and object-GBM models. First, due to the Covid-19 pandemic and associated lockdown in Accra from March 30^th^ to April 20^th^ 2020, we excluded images and pollution data from March 23^rd^ to May 11^th^ 2020, when we were unable to attend to the regular maintenance of monitoring hardware, and therefore data collection was incomplete and uneven across sites.

A small number of cameras experienced internal failure of their clocks, resetting to a factory default of January 2017 at the start of their deployment, which led to images recorded with incorrect timestamps. We corrected the timestamps for these images by re-assigning the initial timestamp based on the start of the monitoring period, which was recorded on a log-sheet when visiting every site. Since each image thereafter was captured at regular five minute intervals, subsequent images were assigned time stamps at five minute intervals. Finally, a small fraction (<1%) of images and pollution data were corrupted and hence unreadable by code. These data were excluded.

Images and pollution data were combined by assigning each image the pollution observation nearest in time, with a requirement that the pollution value was recorded within +/- 30 s of image capture. Where two cameras were placed at a site, both images are assigned pollution data based on this procedure. Some images did not have corresponding pollution values due to a lack of measurements when monitors failed or were unstable. For noise, 83-98% of fixed site images and 99% of rotating site images were assigned pollution data. For PM_2.5_, 68-89% of fixed site images and 79% of rotating site images were assigned pollution data. Full details are provided in Appendix Figure C. As mentioned in section 3.3 we applied a previously developed object detection CNN to all 2.1 million images in our unmerged data set to obtain information on the counts of different object categories within each image, which are used as numerical inputs for our GBM models. Examples of the detected objects within our images may be seen in Appendix Figure D.

1.6 million images were assigned corresponding PM_2.5_ values and 1.9 million images with noise values. Each dataset was divided into training, validation and test sets, as shown in Figure 1. The test set was 10% of all data. Training and validation sets (a 25% holdout of the remainder of data) were used to train the model and set hyperparameters. After determining hyperparameters, the final models were trained on the combined training and validation sets and evaluated on the test set. Both CNN and GBM models used the exact same sets of images, with the former using the entire image and the latter the counts of objects in each image.

### 3.5 Model training

#### 3.5.1 End-to-end CNN

We used a Residual Network with 101 layers (ResNeXt 101) (Xie et al., 2017) as the convolutional neural network (CNN) architecture for classifying noise and PM_2.5_ levels from an entire image. The algorithm was implemented and trained in PyTorch (Paszke et al., 2019) and was pretrained on ImageNet data to enable the CNN to recognise low level features, e.g., edges, which improved model performance, as seen in other computer vision tasks (Huh et al., 2016).

During training, CNNs were given images resized to 224 × 224 pixels with layer depths of 3 to accommodate the Red, Green and Blue channels, with Z-score normalisation applied across all images to assist with the gradient descent process of learning (LeCun et al., 2012). A modified cross-entropy loss function with a log-barrier constraint (Belharbi et al., 2020) was used to account for the ordinal nature of pollution classes, as described in Appendix B. Training was performed on two NVIDIA Quadro RTX 6000 GPUs (48GB memory), with models taking approximately 1 h per epoch and lasted for 30 epochs. The batch size was 32 images, using stochastic gradient descent with an initial learning rate of 0.001, a momentum of 0.9 and a step size of 40. Final models were those which performed best on the validation set during the training process, ranging from the 16-25th epoch.

At training time, data augmentation was used to improve model generalisability and mitigate overfitting to the data (Shorten and Khoshgoftaar, 2019), by uniformly, randomly cropping the image borders, with the central 90% area of the image always preserved, random rotations of the image between 10° anti-clockwise to 10° clockwise, and evenly random flipping of the image in the horizontal plane. These transformations correspond to the variance seen between camera images and the placement at different sites, which had different fields of view and camera orientations.

#### 3.5.2 Object-based gradient boosted machines

We used gradient boosting machines or GBMs as the algorithm for classifying noise and PM_2.5_ from specific, interpretable features, which were counts of objects detected within each image from a separate CNN (Nathvani et al., 2022). GBMs are ensemble tree-based models which use “boosting”, i.e. adaptively changing the weights of data points in the training distribution during the learning process to improve performance on less easily predicted data (Friedman, 2001), which were implemented XGBoost (Chen and Guestrin, 2016) in Python and Scikit-Learn (Pedregosa et al., 2011). GBMs have high efficacy across many problem domains with structured data inputs, due to their ability to learn non-linear relationships between features with robustness to outliers in a flexible and scalable manner (Chen and Guestrin, 2016). They offer advantages compared to linear models, which are more biased in complex data domains, and computationally expensive models such as support vector machines, and artificial neural networks which are typically more cumbersome to optimise. Furthermore, in a preliminary analysis, GBMs had better performance, as measured by classification accuracy, than comparable tree-based methods such as decision trees and random forests.

The input to the GBM models were vectors representing the counts of different objects in each image, e.g., (cars: 2, people: 3, umbrellas: 0...). The model hyperparameters were determined with Bayesian optimisation, with the validation set being used for fixed sites’ data and with 3-fold cross validation when training on 9 folds of rotating site data (Figure 1) and a cross-entropy loss function. The search range for the parameters is given in Appendix Table D. Training was performed during the Bayesian hyperparameter tuning process and was stopped when 5 iterations of tuning yielded no further improvements in overall class prediction accuracy on the independent validation set.

### 3.6 Model evaluation

We compared the performance of both end-to-end and feature-driven approaches to infer plausible contributors to how they predict pollution. We calculated classification accuracy for exact class prediction as well as for when the model classified into the ground truth or adjacent class (shown as “same and ±1 class accuracy”). We evaluated all our models against a null model which measures whether the models do better than simply taking the average from a distribution of training data. We also calculated the models’ accuracy for specific subsets of data under different environmental conditions, including day and night time, and the dry and dusty Harmattan season (November–February). During the Harmattan season dust from the Saharan Desert is carried by trade winds (Adetunji et al., 1979), and there is haze and “redness”(Adetunji et al., 1979; Anuforom, 2007; Ette and Olorode, 1988; McTainsh, 1980; Ochei and Adenola, 2018; Pinker et al., 1994), caused by absorption and scattering of light (Groblicki et al., 1981; Waggoner and Weiss, 1980) and the dust itself, which has a red-brown colour (Breuning-Madsen and Awadzi, 2005; Lafon et al., 2004). These changes in visibility can inform air pollution estimation (Hyslop, 2009; Ozkaynak et al., 1985).

For GBM models in 1a, we quantified the importance of each object for prediction via its permutation importance, which calculates the reduction in the model’s accuracy on the test set, before and after randomly shuffling the values of an input feature (in our case, counts in a given object category) across images. Object counts in our data were correlated among different object categories across images (Nathvani et al., 2022), e.g., people and cars. Therefore a given object’s importance score might be lower when multiple objects are used for prediction than when single objects are used, because other correlated objects capture some of the same information.

## 4 Results and Discussion

**Figure 3:**
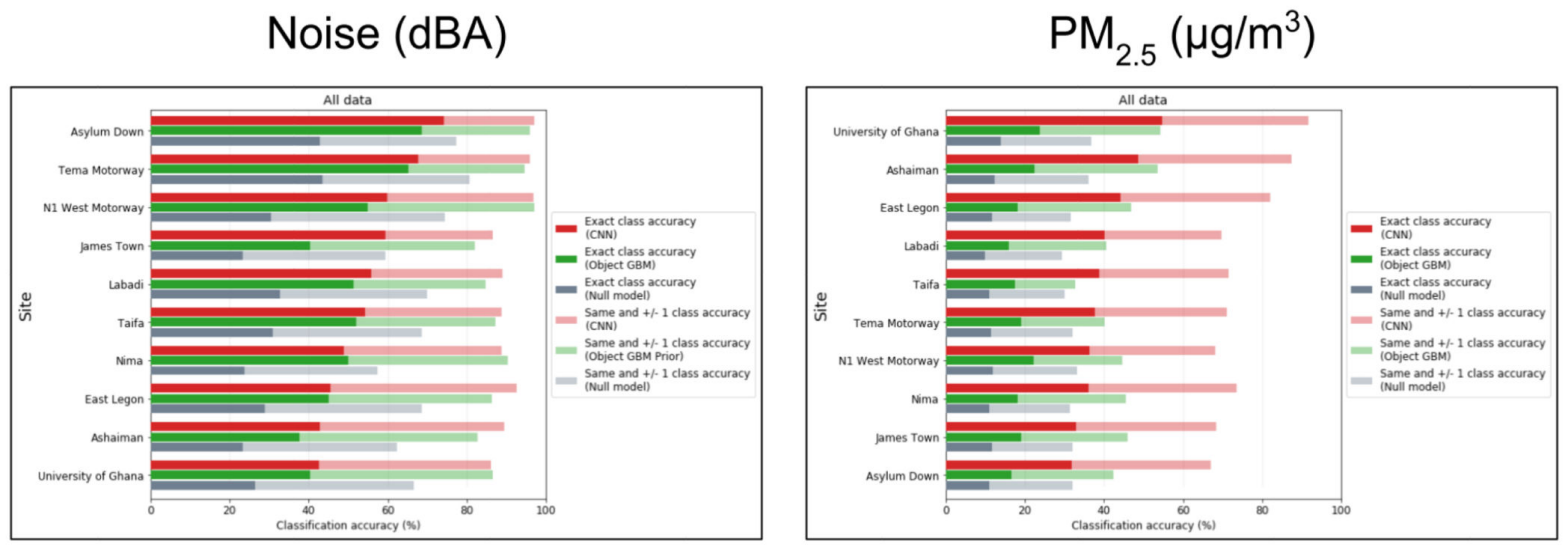
The classification accuracy achieved by CNN and GBM models trained and tested on images from the same fixed site (Question 1a) is shown for noise and PM_2.5_.

For noise, classification accuracy at different times in the same location (i.e. Question 1a) for both the end-to-end (CNN) and object-based (GBM) models ranged from 40 to 70% across sites, considerably outperforming their null models (Figure 3). Accuracy increased to 80-90% for neighbouring (±1) class classification. The performance of the two models was similar, with CNNs slightly outperforming GBM models. Predictions at sites with high road-traffic, such as Asylum Down, Tema Motorway and N1 West Motorway, had higher accuracy than those at other sites. Noise predictions using CNN models were often more accurate in the daytime (59.9% average classification accuracy across all sites) than night time (49.8%) (Appendix Figure E) which may result from predictive features such as people, traffic and marketplace indicators (e.g., umbrellas) being present, and more visible in the day, as in Appendix Figure D, since street lighting conditions vary across our sites.

PM_2.5_ classification had lower accuracy than that of noise in most model and site combinations (Figure 3). There was also a larger discrepancy between the predictive performance of CNN and GBM models for PM_2.5_, with CNN models achieving 30-55% classification accuracy and GBM models 15-25%, though both outperformed null model benchmarks. Sites with higher classification accuracy for noise performed less well for air, and vice versa; for example, the three poorest performing sites for noise (University of Ghana, Ashaiman and East Legon) had the greatest accuracy for CNN models when predicting PM_2.5_. Accuracy of CNN models reached 70-90% for neighbouring class classification, and that of GBM models 30-50%. Unlike noise, performance of PM_2.5_ classification using CNN models differed little between day (40.2% average classification accuracy across all sites) and night time (39.6%) and had higher accuracy during the Harmattan period (57.7%) than in other times (35.1%) (Appendix Figure E), whereas GBM models had no consistent advantage during the Harmattan (17.5%) than in other times (19.9%).

**Figure 4:**
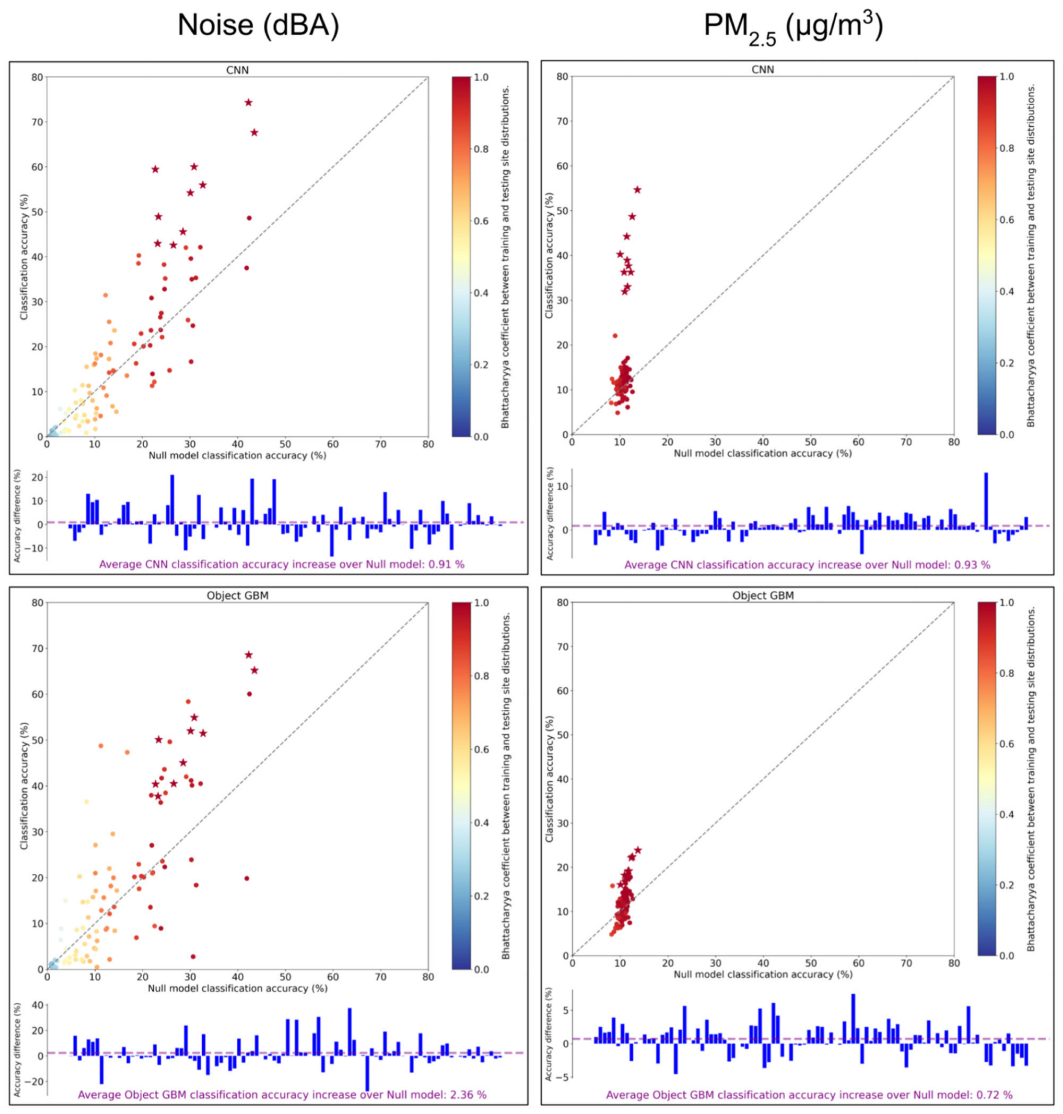
The classification accuracy achieved by the CNN (left) and GBM (right) models trained and tested from images at one fixed site and tested at a different fixed site (Question 1b) for both noise (top) and PM_2.5_ (bottom) prediction. Points are coloured by the Bhattacharya coefficient between the pollution distributions between the training and testing sites, which is a measure of the overlap between the distributions. Data points with a star indicate testing and training performed at the same site, as in 1a. All null models are from the fixed site used for training. Below each scatterplot the relative improvements in classification accuracy over the null model accuracy is given for each data point (i.e. the vertical distance between the round points and the dashed diagonal line in the scatterplot); the purple dashed line shows the average across all data points, illustrating whether models achieved improvement over their benchmarks overall.

When trained at one fixed site and tested at a different fixed site (Question 1b), accuracy dropped compared with same-site testing and CNN models for noise and PM_2.5_, performed similar to null model benchmarks, demonstrating the inability of models to generalise from the measurements at a single site (Figure 4). For noise, the variation in accuracy of GBM models was greater than that of CNNs, but on average achieved greater improvement over null model benchmarks (+2.4%) than did CNNs (+0.9%). For PM_2.5_ accuracy ranged 7-20% for both GBM and CNN models, with the latter achieving greater improvement over the null model benchmarks (+0.9%) than did GBMs (+0.7%). In all cases, accuracy and null model performance broadly increased with Bhattacharya coefficient, a measure of the similarity of pollution distributions between training and testing site (Bhattacharyya, 1943).

**Figure 5:**
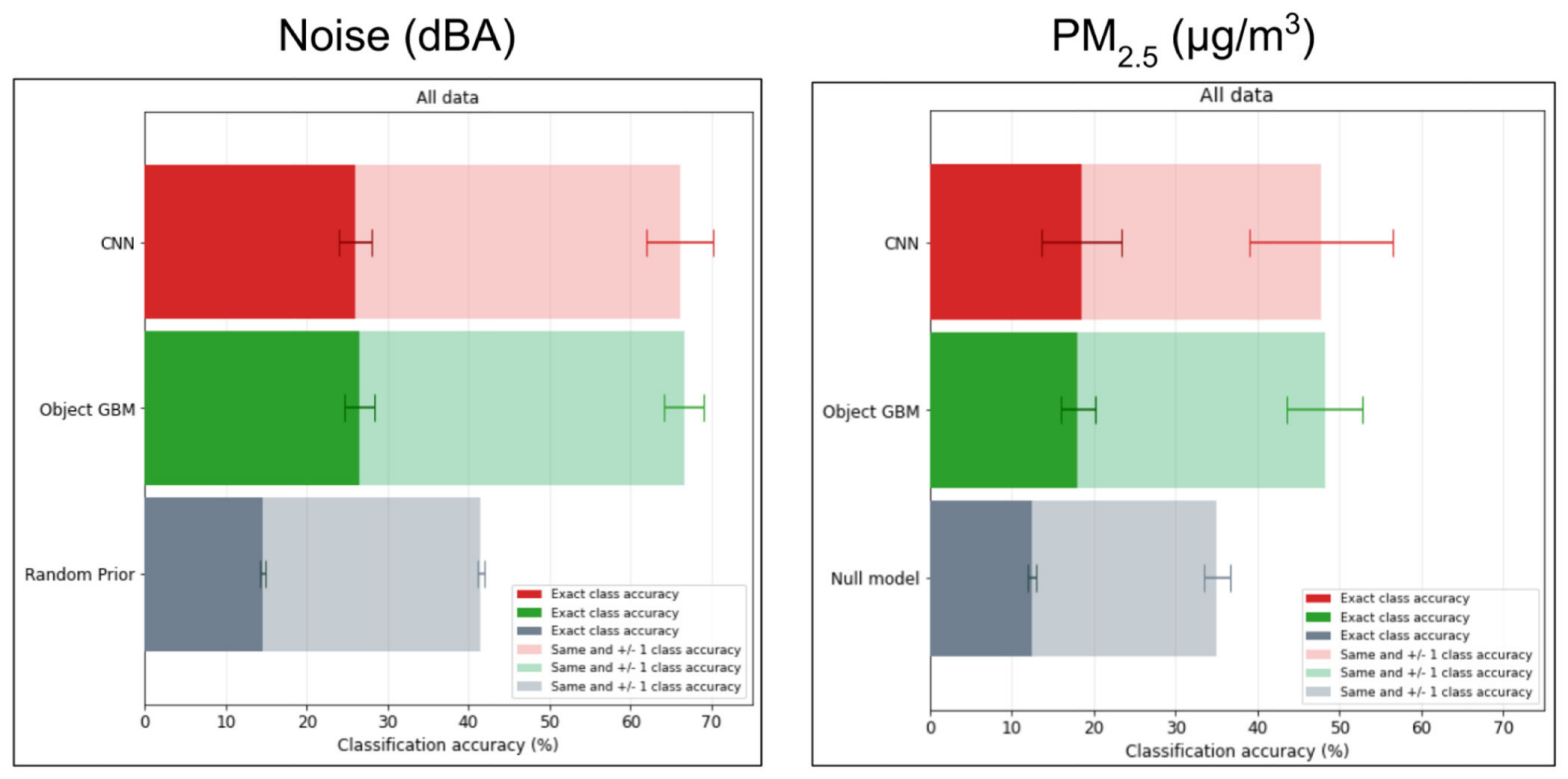
Classification accuracy achieved by CNN and GBM models trained and tested on images from rotating sites (Question 2b) for both noise and PM_2.5_ prediction. Accuracies are shown as the average over the folds of training data, as shown in Figure 1. The bars show the standard deviation of the accuracy across different folds.

Training on nine fixed sites with abundant (~1,000,000) data (Question 2a) produced similar results for generalising to a single, unseen fixed site as for models trained at single fixed sites (Appendix Figure F). For noise, accuracies were at best 30-40% for both CNN and GBM models, and in some instances similar to the null model; neither CNN or GBM models had a distinct advantage. For PM_2.5_, both CNN and GBM model accuracy remained similar to null model performance. CNN and GBM models trained using fewer images (~100,000) from ~90% of rotating sites (121-122 sites) for classifying noise and PM_2.5_ at the remaining rotating sites (Questions 2b) outperformed their respective null models (Figure 5). As in temporal transferability (Question 1a), models performed better in classifying noise than PM_2.5_, but the advantage of CNN over GBM models disappeared. Noise models had 25% accuracy for exact class and 65% when allowing for neighbouring class classification, with little variance (± 2.5%) between folds, whilst PM_2.5_ models had 17.5% accuracy, and 47.5% allowing for neighbouring class classification. For noise, but not for PM_2.5_, CNN accuracy for daytime images was significantly higher than on night time images (Appendix Figure G). CNN models had similar performance across all land use categories for both forms of pollution (Appendix Figure G).

When comparing the approaches in Questions 2a (fewer sites with more data per sites) and 2b (more sites with fewer data per sites) with consistent test data (Question 2c), models trained on a smaller amount of data from many (rotating) sites performed better than those trained on many times more data from a smaller number of (fixed) sites, for both noise and air pollution models (Appendix Figure H). This suggests that with finite monitoring capacity, a diversity of locations for data gathering is more likely to produce spatial generalisability for pollution prediction using CNN models than long-term capture of data at fewer locations, highlighting the importance of optimising the spatial as well as temporal representativeness of data within cities for pollution modelling, as seen in other domains of computer vision (Schat et al., 2020).

### 4.1 Object feature importance

**Figure 6:**
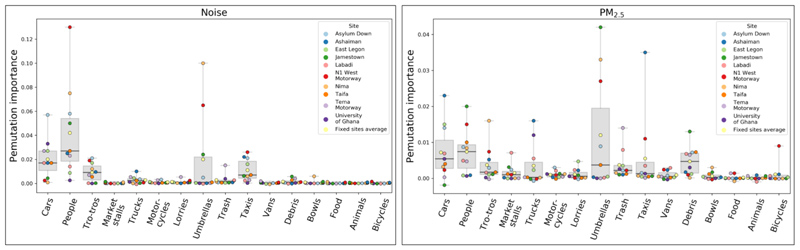
Permutation importance for each object used as inputs to the noise and PM_2.5_ GBM models in Question 1a, for each fixed site. Permutation importance is calculated on the test set for each model, as shown in Figure 1.

In GBM models developed in 1a, cars, people, taxis, umbrellas and tro-tros contributed most to predictions for both noise and PM_2.5_ (Figure 6). For PM_2.5_, debris and trucks also contributed to prediction accuracy. These object categories were frequently detected in fixed site images. We calculated the Spearman correlation between object counts and noise and PM_2.5_ levels across images at each fixed site (Appendix Table E) in order to test whether this correlation explained an object’s permutation importance. The explained variance, calculated as the square of Pearson correlation between object-pollution correlations and the permutation importance for each object was 0.86 for noise and 0.76 for PM_2.5_. Heuristically, the greater the correlation of an objects’ counts with that of pollution, the greater its contribution to model prediction accuracy, which may also indicate why noise models in 1a performed better than those for PM_2.5_. Since objects visible in the images had greater impact on the accuracy of noise prediction compared to PM_2.5_, CNN models for noise may have learned to rely on the same features as those used by the GBM models prediction. This may in turn explain the similar performance between the two models in 1a, which we further examine in the section below.

As shown in Figure 6, the objects with the highest permutation importance were various types of vehicles, consistent with previous research on the significant contributions from road traffic to both air and noise pollution (Dionisio et al., 2010; Onuu, 2000; Rooney et al., 2012). In addition, umbrellas were frequently present in images due to their extended use in the daytime to protect market vendors and their merchandise from the sun and rain. Markets also attract high levels of vehicular traffic (Agyapong and Ojo, 2018), people (Asante, 2020; Asante and Mills, 2020), and roadside cooking and food vending, which collectively increase noise and air pollution (Alli et al., 2021; Clark et al., 2021). Furthermore, for PM_2.5_ models, debris had higher than average feature importance. Although not a source, debris is more visible and readily detected by our object detection algorithm during daylight hours, serving as a proxy for time of day, and for sites with diurnal patterns of PM_2.5_. In addition, debris may be more visible when unobscured by other objects, acting as an implicit indicator for a lack of crowds or traffic, and instances where the road surface, from which dust particles may be resuspended, is exposed.

### 4.2 Harmattan influence

To probe why CNN models in 1a performed better during the Harmattan season, when PM_2.5_ levels were much higher, we derived characteristics of images related to changes in hue and haze between Harmattan and non-Harmattan periods, since previous work has demonstrated that changes due to Harmattan dust, such as an increase in “redness” and haze are indicators and predictive factors for pollution (Adetunji et al., 1979; Anuforom, 2007; Ette and Olorode, 1988; McTainsh, 1980; Ochei and Adenola, 2018; Pinker et al., 1994), as well as light scattering from dust (Groblicki et al., 1981; Waggoner and Weiss, 1980). Other approaches for predicting pollution from images have also used these features (Feng et al. 2021; Wang et al. 2022; Liu et al. 2015; Ganji et al, 2020) and we therefore created feature metrics which relate to qualities of hue and haze in our images in order to study our models. For daytime images, we compared mean pixel intensity in each colour channel between these periods: red, green and blue. For night-time images in single-channel grayscale, we used mean pixel intensity and pixel intensity standard deviation (SD). Red pixel intensity was greater during the Harmattan period, whilst the opposite was seen for blue (Appendix Figure I). Furthermore, night-time pixel intensity was higher at eight of ten sites during Harmattan, while pixel SD was lower at seven sites. To infer to what extent this information was used by our CNN models for PM_2.5_ classification, we calculated Spearman correlations between mean red and blue pixel intensity and each image’s associated PM_2.5_ value (Appendix Table F). The average across sites was 0.11 for red pixel intensity and -0.21 for blue pixel intensity. Similarly, grayscale pixel intensity tended to be positively correlated with air pollution, 0.10, whilst grayscale pixel SD was negatively correlated, -0.18, consistent with light sources appearing more diffuse in hazy conditions due to light scattering.

The magnitude of these correlations was also greater during the Harmattan period, during which CNN models in 1a had greater accuracy (Appendix Table F). The Pearson correlation across all 10 sites between the aforementioned Spearman correlations for red pixel intensity and the accuracy of a CNN model at that site in 1a, was 0.73. In heuristic terms, the greater the correspondence between redness of image and air pollution, the better the model performed on average.

### 4.3 Model interpretability across time and space

Our results suggest that specific features, such as the objects selected for the GBM model, are better attuned at predicting noise, whilst predictive performance for PM_2.5_ is somewhat improved by leveraging more complex visual features from images such as red pixel channel intensity and haziness. This is supported by the discrepancy between day and night image accuracy for CNN models for noise in 1a and 2b, which may result from objects being more visible and present in the day than night, allowing CNN models to make more accurate predictions of noise, but not PM_2.5_, using daytime images.

**Figure 7:**
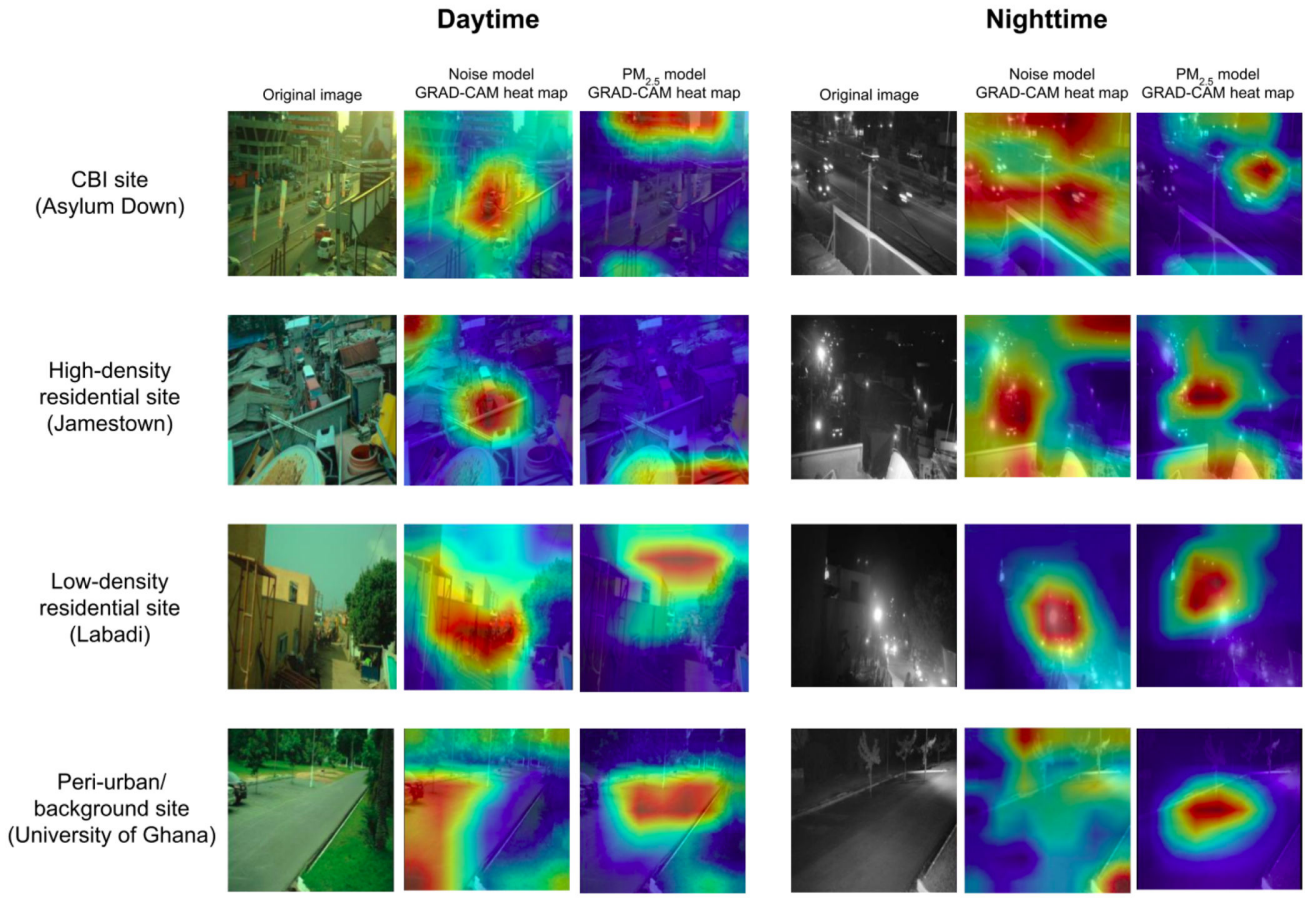
Grad-CAM visualisation heat maps for four fixed sites of different land-use categories, obtained from CNN models. The highlighted regions in each image (red) indicate the features most salient to the model’s prediction, for that image. The same image is shown as used by the corresponding noise and PM_2.5_ models, developed in Question 1a.

These observations also suggest that features learned by the CNN for noise classification are likely similar to the objects used by the GBM models, whilst being of less importance for CNN models for PM_2.5_. To investigate this, we generated a sample of gradient class activation maps (Selvaraju et al., 2017) (Grad-CAMs) for our CNN models in 1a. Grad-CAMs use gradient descent to work backwards from a network’s class prediction from a given image to the regions in the image itself which most contributed to that prediction. Figure 7 shows that noise models focus either on visible objects or the location of main thoroughfares, whilst PM_2.5_ models tend to focus on either fixed features of the built environment, or the sky, supporting the possibility of the object-driven nature of noise models and the reliance of PM_2.5_ CNN models on complex visual features. This may be due to the fact that noise is transitory and spatially linked to sources or their proxies such as market stalls and their associated umbrellas. By contrast, PM_2.5_ persists at a location longer than its sources, and is composed of both local (e.g., traffic) and non-local (dust, neighbouring regions’ emissions) sources.

## 5 Conclusions

We used a unique dataset of co-located images and pollution measurements, and two different modelling approaches, to investigate how images predict spatially and temporally resolved noise and air pollution measurements in a major city in Africa. Most models developed in this work surpassed null model accuracy baselines, indicating that models can learn information latent to images, beyond extrapolation from outcome data. Models had similar performance across different land-use categories within the city and across both day and night time, with a slight advantage to model accuracy in the daytime for noise. However, even when training and testing models at single sites, classifying noise and PM_2.5_ with either CNN or GBM models had moderate performance. No model surpassed 80% classification accuracy, and performance was considerably lower when testing on previously unseen locations. This may be due to a combination of factors including the static nature of images, which may fail to capture transitory sources, e.g., emergency vehicle sirens, compared with exposures which are averaged over one minute’s observation. Furthermore some pollution sources (and predictors) are non-local or out of the field of view of the corresponding image’s camera. Since the models developed in questions 1b to 2c rely on a single images from consumer-grade digital cameras to predict pollution in an unseen location, our dataset and methodology also informs on the viability of pollution modelling from comparable camera technology, including CCTV networks which are increasingly deployed in African cities and mobile phone camera capture, as has been used elsewhere in the literature. In addition, we intentionally tested models under the challenging condition of estimating pollution from a single image in time and space, in order to independently and conservatively assess the additional benefit images may confer above data extrapolation. Future work could improve model accuracy by making use of CNN architectures with multiple image inputs across time, or in the case of feature-driven prediction, with object counts across a series of time-points prior to the moment in time whose pollution levels are estimated. Similarly, image and/or object counts from neighbouring sites might also help predict pollution at a given location. These may prove especially beneficial for the prediction of PM_2.5_, whose presence is more persistent than many of its transitory sources, such as travelling cars.

Where pollution sources are complex and vary widely across small spatial scales, the inclusion of data from many sites improved the spatial generalisability of CNN models in comparison to an abundance of data from a small number of locations. Our results highlight the importance of optimising the spatial as well as temporal representativeness of data gathered within cities for pollution modelling, as seen in other domains of computer vision. Although spatially representative data improved model performance, for CNNs model accuracy also varied under particular times of day and seasons, related to time-varying environmental factors. We also find that models are capable of making comparably accurate estimates for nighttime air and noise pollution, particularly for PM_2.5_ estimation, when such data is gathered and used for modelling, alongside corresponding images. This shows the need for temporally diverse, paired pollution and image data that capture urban environmental change on both short (< 1 h) and long (~1 year) timescales.

Overall, our results show that inference of noise and PM_2.5_ from imagery is a feasible but challenging task, especially as the spatial and temporal scale of prediction becomes smaller, which is relevant for detailed policy formulation, such as dynamic smart congestion pricing, and evaluation of impacts on human exposure. Therefore, accurate and generalisable estimates of short timescale pollution in cities continues to require primary data collection at representative and diverse scales to support these efforts.

## Supplementary Material

Appendix

## Data Availability

Our analysis code, trained models, object count data and site metadata can be downloaded from http://globalenvhealth.org/code-data-download/ and http://equitablehealthycities.org/data-download/ upon publication of the paper. Requests for re-analysis of images should be sent to the corresponding authors.
