# Supplementary material for: Beyond here and now: evaluating pollution estimation across space and time from street view images with deep learning
Source: Sci Total Environ. Author manuscript; Available in PMC 2023 Dec 1. (PMC7615099; doi:10.1016/j.scitotenv.2023.166168)
Supplement: Appendix [file EMS187575-supplement-Appendix.docx]

Beyond here and now: evaluating pollution estimation across space and time from street view images with deep learning - APPENDIX

*Ricky Nathvani^*†1,2^, Vishwanath D^†1,2^, Sierra N. Clark^1,2^, Abosede S. Alli^3^, Emily Muller^1,2^, Henri Coste^1,2^, James E Bennett^1,2^, James Nimo^4^, Josephine Bedford Moses^4^, Solomon Baah^4^, Allison Hughes^4^, Esra Suel^1,2,5^, Antje Barbara Metzler^1,2^, Theo Rashid^1,2^, Michael Brauer^6^, Jill Baumgartner^7,8^, George Owusu^9^, Samuel Agyei-Mensah^10^, Raphael E. Arku^‡3^, Majid Ezzati^‡1,2,11^*

** Corresponding author: r.nathvani@imperial.ac.uk*

*† Joint first authors*

*‡ Joint senior authors*

1 Department of Epidemiology and Biostatistics, School of Public Health, Imperial College London, London, UK

2 MRC Centre for Environment and Health, School of Public Health, Imperial College London, London, UK

3 Department of Environmental Health Sciences, School of Public Health and Health Sciences, University of Massachusetts, Amherst, USA

4 Department of Physics, University of Ghana, Accra, Ghana

5 Centre for Advanced Spatial Analysis, University College London, London, UK

6 School of Population and Public Health, University of British Columbia, Vancouver, Canada

7 Institute for Health and Social Policy, McGill University, Montreal, Canada

8 Department of Epidemiology, Biostatistics, and Occupational Health, McGill University, Montreal, Canada

9 Institute of Statistical, Social & Economic Research, University of Ghana, Accra, Ghana

10 Department of Geography and Resource Development, University of Ghana, Accra, Ghana

11 Regional Institute for Population Studies, University of Ghana, Accra, Ghana

**Content**

**Appendix A.** Data collection campaign. ………………………………………………………………... 4 **Appendix B.** Ordinal loss function ……………………………………………………………………... 6

**Appendix C.** Regression model sensitivity analysis. …………………………………………………... 8

**Appendix Table A.** Comparison of our study with previous studies predicting pollution from images .10

**Appendix Table B.** Characteristics of fixed sites and rotating site folds.……………………………… 12

**Appendix Table C.** Performance of the final object detection model. ……………………………….... 14

**Appendix Table D.** Hyperparameter search range for object models. ………………………………….16

**Appendix Table E.** Spearman correlations between object counts and pollution. …………………….. 17

**Appendix Table F.** Correlation of image characteristics with PM_2.5_. …………………………………. 18

**Appendix Figure A.** Location of measurement sites. ...……………….………………………………...20

**Appendix Figure B.** Noise and PM_2.5_ distributions across sites and folds. ….…………………………. 21

**Appendix Figure C.** Overlap of pollution and image data. ……………………………………………. . 23

**Appendix Figure D.** Objects identified in example images. ………………….………………………… 24

**Appendix Figure E.** Fixed site model accuracy by time of day and Harmattan season. (Question 1a) .. 25

**Appendix Figure F.**  Multiple fixed sites model accuracy (Question 2a) ……………………………… 26

**Appendix Figure G.**  Multiple rotating sites model accuracy (Question 2b) …………………………... 27

**Appendix Figure H.** Comparison of fixed vs rotating sites model accuracy…….……………………. 28

**Appendix Figure I.** Descriptive characteristics of images during Harmattan and non-Harmattan.…….. 29

**Appendix References** …………………………………………………………………………………... 31

**Appendix A.** Data collection campaign.

Accra is the largest city and capital of Ghana and amongst the largest in SSA, acting as a hub for business, technology and education^1,2^, with a population of five million^3^. The GAMA comprises a central urban core, the Accra Metropolitan Area (AMA, ~2 million people), other metropolises and municipalities, and peri-urban and largely rural areas in the periphery. We collected data at 135 sites that represent a variety of socioeconomic status, land use, road types and microenvironments within the Greater Accra Metropolitan Area (GAMA ~1,500 km^2^). The locations of the 135 rotating sites were selected through stratified random sampling from a dataset representing four land cover and neighbourhood classes of Accra defined as the following: formal, mostly low- and medium-density, residential areas; informal, mostly high-density, settlements and slums; industrial and commercial or business areas; and “other” areas that were largely peri-urban or rural, and have relatively dense vegetation (i.e., forest, grassland), barren land (i.e., sand, soil, dirt) or water. The locations of measurement sites are shown in Appendix Figure A, with the land-use classes of each of our fixed sites given in Appendix Table B for reference. The AMA was oversampled due to its relative high population density. At each of these sites, camera imagery, noise and PM_2.5_ measurements were recorded concurrently. Further details regarding the nature of our sites may be found in our study protocol^4^.

Each site, situated on a street, had a monitoring station placed ~4m off the ground with one or two time-lapsed cameras capturing full colour images at five minute intervals. Images were taken by automatically programmed Moultrie-50 camera traps at regular five minute intervals, which allowed each camera to store a week’s worth of data. Images were taken at 20 Megapixels resolution (1960 x 1080 pixels), with a 36.7° field of view. Night time images (~6pm to ~6am) were captured in grayscale when brightness levels drop below a threshold detected by the camera, at which point images are taken with an infrared flash. Each camera recorded the timestamp for captured images, which we retrieved from metadata for our work. Cameras were placed in protective, orientable cases aimed at main public thoroughfares as identified by the authors. 90 of our rotating sites hosted two cameras, while the remaining 45 hosted one camera, based on how broad the field of view of interest (the street thoroughfare) was.

Continuous PM_2.5_ concentrations were measured with low-cost sensors (Zefan monitors). The Zefan uses light scattering technique via the Plantower sensor^15^ (model PMS7003) to record average concentrations of PM_2.5_ in µg/m^3^ at 1-minute intervals. To improve the accuracy of these measurements, data were calibrated against weekly integrated gravimetric measurement from a UPAD monitor, details of which may be found in our study protocol^4^ and in a paper on PM_2.5_ variations throughout the city^4,5^. Sound level (A-weighted decibels (dBA)) were measured with the Noise Sentry Sound Level Meter (NSRT_mk3) from Convergence Instruments^4^ (Canada, Quebec). The Sound Level Meters recorded and integrated equivalent continuous sound levels (LAeq1min dBA) every 1 minute. Details of measurement methods and quality assurance and control can be found in our study protocol^4^. The sensors were positioned next to the cameras and recorded integrated 1-minute measurements of noise levels (averaging over the recorded levels in each 1 minute interval). The distributions of noise and air pollution data in the data sets compiled as part of our study design are shown in Appendix Figure B.

**Appendix B.** Ordinal loss function

In many CNN classification tasks, a cross-entropy loss function is used during training to penalise incorrectly assigned classes. However, this loss does not account for the structure of classes with respect to one another, as in classification problems such as ours, where the classes have an inherent ordinal structure. i.e. the ordering of classes matters and neighbouring classes are meaningfully more similar to one another as compared with distant classes.

We therefore tested both a standard cross-entropy loss function for classification, as well as an approach developed by Belharbi et al^11^. The latter incurs a greater penalty the larger the distance between the predicted and true class, by treating classification as a constrained optimisation formulation in which the loss function is unimodal on the true class and consistent with the order of classes. This is performed with an extension to the standard cross-entropy term in the loss function in the form of a log-barrier^11^.

For a set of training data $\left\{ \left( X^{i}, y^{i} \right) \right\}_{i=1}^{n}$ where $X^{i}$ are input images and $y^{i}$ is one of $c$ ordinal classes, $y_{1}<y_{2}< ... y_{c-1}<y_{c}$, such as our pollution categories, the posterior probability from our CNN model function $M(X; \theta)$ (where $\theta$ are the parameters of the network) for a given class, $k$, is computed with a standard softmax function applied to the logit scores, $s$, produced by the CNN’s final layer: $\hat{p}\left( k|X \right) =exp(s_{k}) / \sum_{j=1}^{c} exp(s_{j})$. As well as the standard cross entropy loss, $C\left( M(X,\theta\right), y) = -log\left( \hat{p}\left( y|X \right) \right)$, the log barrier terms, $\hat{I}$, proposed by Belharbi et al^11^ were included in the training process:

$\min_{\theta}C\left( M(X,\theta\right), y) + \left( \sum_{j=1}^{y-1} c\left( \Delta_{k}^{k+1}(s) \right) + \sum_{j=y}^{c-1} \hat{I}\left( \Delta_{k+1}^{k}(s) \right) \right)$,

where $\Delta_{m}^{l}(s) = s_{m}- s_{l}$, and the log barrier takes the form:

$\hat{I} = -\frac{1}{t}log(-r) if r \leq\frac{1}{-t^{2}} , (t\times r) -\frac{1}{t}log(\frac{1}{t^{2}}) +\frac{1}{t} otherwise$, where $t$ is a temperature variable that is slowly increased by a constant factor every epoch of training. During the comparison of regressors and classifiers described in Appendix C, we performed training with and without the log-barrier extension method and observed that validation set classification accuracy was improved when included. Therefore the log-barrier extension was used when training all final CNN models.

**Appendix C.** Regression model sensitivity analysis.

Our task of predicting noise and PM_2.5_ at specific time point from an image could be formulated either as a regression task, to predict a continuous value estimate for noise in A-weighted decibels (dBA) and PM_2.5_ concentrations in micrograms per metre-cubed (μ/m^3^), or as a classification problem, to predicted membership of a pollution value within one of mutually exclusive classes. We opted for the latter for interpretability reasons, as stated in the main paper, and also based on a preliminary analysis during the study design phase, outlined below.

For PM_2.5_ and noise, we constructed both a regressor and classifier model for both CNN and object GBM models, based on data at two of fixed sites, Asylum Down and Jamestown (Appendix Figure B). For the CNN, the same learning rates, batch sizes and number of training epochs were used for the regression and classifer models, with the regressors being trained with a Mean Squared Error (MSE) loss function, and the classifier with the Ordinal Classification Loss accuracy loss function described in the previous section. For object GBM models, an identical hyperparameter selection process (described in the previous section) was implemented, with MSE loss used for regressors and total classification accuracy used as a loss function for classifier models.

To compare regressors against classifiers, we converted the continuous estimates from the regressors into the corresponding classification category, e.g. a 58 dBA estimate put into the class 55-<60 dBA, to compare equivalent total classification loss accuracy for all models. In all cases, classifiers achieved a higher total classification loss (the average of accuracy achieved across all classes), and were hence adopted for our analysis.

| Study | Exposure(s) | Study location | Pollution estimate | Image temporal resolution | Additional model inputs | Range and number of images | Time between image and pollution estimate | Modelling approach | Outcome or feature driven |
| --- | --- | --- | --- | --- | --- | --- | --- | --- | --- |
| Liu et al (2016) | PM_2.5_ | Beijing, Shanghai (China) and Phoenix (US) | City-wide | Hourly | None | 1 year (6587) | < 1 hour | Regression | Feature |
| Chakma et al (2017) | PM_2.5_ | Beijing, China | City-wide | Single instance | None | 4 years (591) | < 1 hour | Classification | Feature |
| Gu et al (2017) | PM_2.5_ | Beijing, China | City-wide | ~ 1.5 days | None | 3 years (750) | < 1 hour | Regression | Feature |
| Zhang et al (2018) | PM_2.5_, PM_10_ | Beijing, China | City-wide | Single instance | None | 1 year (36,624) | < 1 hour | Classification | Outcome |
| Feng et al (2021) | PM_2.5_ | Beijing, China | City-wide | 30 minutes | None | 11 months (13,000) | < 1 hour | Regression | Feature |
| Wang et al (2022) | PM_2.5_ | Beijing, China | City-wide | Daily | None | 1 year (1954) | < 1 hour | Regression | Feature |
|  |  |  |  |  |  |  |  |  |  |
| Liu et al (2015) | PM_2.5_ | Beijing, China | Local | Daily | None | 6 months (200) | < 1 day | Regression | Feature |
| Ganji et al (2020) | UFP, BC | Toronto, Canada | Local | Single instance | Aerial imagery | 3 months (3471) | < 5 years | Regression | Feature |
| Hong et al (2020) | UFP, noise | Montreal, Canada | Local | 5 minutes | Audio, temperature, wind speed | 10 months (154,000) | < 1 minute | Both | Outcome |
| Qi et al (2021) | BC, PN | Blacksburg, USA | Local | Single instance | None | 4 months (52,500) | ~ 1 year | Regression | Feature |
| Won et al (2022) | PM_2.5_, PM_10_ | Seoul, Korea | Local | 1 minute | Humidity, wind speed | 4 months (11,186) | < 1 hour | Regression | Outcome |
| **Our study (2023)** | **PM_2.5_, noise** | **Accra, Ghana** | **Local** | **5 minutes** | **None** | **15 months (2.1 million)** | **< 1 minute** | **Classification** | **Both** |

**Appendix Table A.** Comparison of our study with previous studies predicting pollution from images.

“City-wide” pollution estimates refers to studies where pollution measurements used for modelling are from a single location in the city, with the corresponding location of the images some distance away. “Local” pollution estimates refer to studies where the location of the captured image and the pollution measurements spatially coincide. Outcome and feature driven refer to studies where all model features are learned from images during the model training process, or where specific features are extracted from images for modelling purposes, respectively. UFP, BC and PN refer to ultrafine particles, black carbon and particle number.

| **Site** | **Land use category** | **Number of images merged with sound-level values** | **Number of images merged with PM_2.5_ concentration values** |
| --- | --- | --- | --- |
| Asylum Down | Commercial/business/industrial | 166863 | 166341 |
| Ashaiman | Medium/low-density residential | 88964 | 81411 |
| East Legon | Medium/low-density residential | 166952 | 135845 |
| Jamestown | High-density residential | 85955 | 73698 |
| Labadi | Medium/low-density residential | 90345 | 68244 |
| N1 West Motorway | Commercial/business/industrial | 184958 | 168598 |
| Nima | High-density residential | 187181 | 168306 |
| Taifa | Medium/low-density residential | 157572 | 145042 |
| Tema Motorway | Commercial/business/industrial | 194756 | 171421 |
| University of Ghana | Peri-urban background | 162122 | 128924 |

| **Rotating site fold** | **Number of images merged with sound-level values** | **Number of images merged with PM_2.5_ concentration values** |
| --- | --- | --- |
| Fold 1 | 40983 | 35261 |
| Fold 2 | 40431 | 32107 |
| Fold 3 | 38090 | 31830 |
| Fold 4 | 41067 | 28554 |
| Fold 5 | 40753 | 37387 |
| Fold 6 | 42203 | 25053 |
| Fold 7 | 40674 | 34939 |
| Fold 8 | 41652 | 39222 |
| Fold 9 | 41508 | 34263 |
| Fold 10 | 35944 | 24816 |

**Appendix Table B .** Characteristics of fixed sites and rotating site folds.

Further details on the nature and choice of land use categories may be found in our protocol paper (4).

| Object | Counts in combined training and validation set (1,000 images) | Counts in testing set (250 images) | mAP^‡^ | mAP@0.5^‡^ |
| --- | --- | --- | --- | --- |
| Person | 3,331 | 855 | 0.389 | 0.728 |
| Car | 1,302 | 336 | 0.400 | 0.673 |
| Trash | 826 | 211 | 0.092 | 0.205 |
| Tro tro | 551 | 139 | 0.367 | 0.622 |
| Debris | 510 | 128 | 0.061 | 0.122 |
| Umbrella | 478 | 139 | 0.437 | 0.735 |
| Taxi | 363 | 85 | 0.480 | 0.681 |
| Cooking bowl/pot | 143 | 34 | 0.072 | 0.181 |
| Pick-up truck | 140 | 34 | 0.272 | 0.425 |
| Market stall | 133 | 35 | 0.098 | 0.192 |
| Food | 128 | 32 | 0.064 | 0.165 |
| Motorcycle | 126 | 31 | 0.314 | 0.729 |
| Lorry | 107 | 28 | 0.336 | 0.473 |
| Van | 105 | 26 | 0.044 | 0.088 |
| Street vendor | 97 | 22 | 0 | 0 |
| Animal | 74 | 19 | 0.295 | 0.579 |
| Bicycle | 53 | 13 | 0.195 | 0.375 |
| Bus | 20 | 5 | 0.307 | 0.438 |
| Cookstove | 14 | 4 | 0 | 0 |
| Loudspeaker | 13 | 4 | 0 | 0 |
| Total (frequency weighted) | 8,514 | 2180 | 0.318 | 0.575 |
| Total (average of categories) | NA | NA | 0.211 | 0.370 |

‡ Mean average precision (mAP)

**Appendix Table C.** Performance of the final object detection model.

For each object category, and across all categories combined, the table shows mean average precision (mAP), which measures whether the network accurately identifies both the presence of an object and localises its location and size, as represented by its boundaries. A true positive is defined when the predicted bounding box overlaps with the ground truth box above a range of intersection-over-union (the overlapping area between two bounding boxes divided by the area of their union) thresholds, from 0.50 to 0.95 in 0.05 intervals, and is identified as the correct object category. Identification is defined as when the final layer object classifier produces a confidence score above a range of thresholds for a given object category. Precision is defined as the proportion of bounding boxes that are true positives while recall is the fraction of true-positive detections as a proportion of all ground truth boxes. The average precision is the area under the precision-recall curve, obtained from varying the confidence score thresholds. The mean refers to the mean taken across different intersection-over-union overlap thresholds, and confidence scores either for individual object categories or across object categories. This metric was originally constructed to evaluate performance of diverse models on the MS-COCO dataset. The network was trained on the 1,000 images in the training and validation sets and tested on the 250 images in the test set.

| Hyperparameter | Range | Distribution type |
| --- | --- | --- |
| scale_pos_weight | 10^-6^-500 | Log-uniform |
| reg_lambda | 10^-9^-1000 | Log-uniform |
| learning_rate | 0.01-1 | Log-uniform |
| min_child_weight | 0-5 | Uniform |
| max_depth | 0-50 | Uniform |
| max_delta_step | 0-20 | Uniform |
| subsample | 0.01-1 | Uniform |
| colsample_bytree | 0.01-1 | Uniform |
| colsample_bylevel | 0.01-1 | Uniform |
| reg_alpha | 10^-9^-1 | Log-uniform |
| gamma | 10^-9^-0.5 | Log-uniform |
| n_estimates | 50-100 | Uniform |

**Appendix Table D.** Hyperparameter search range for GBM models.

The range of parameters within which Bayesian Hyperparameter Optimisation was conducted, for our object-based GBM models. Naming conventions follow those as used in XGBoost, with details of each parameter’s definition and function available in the library documentation^14^.


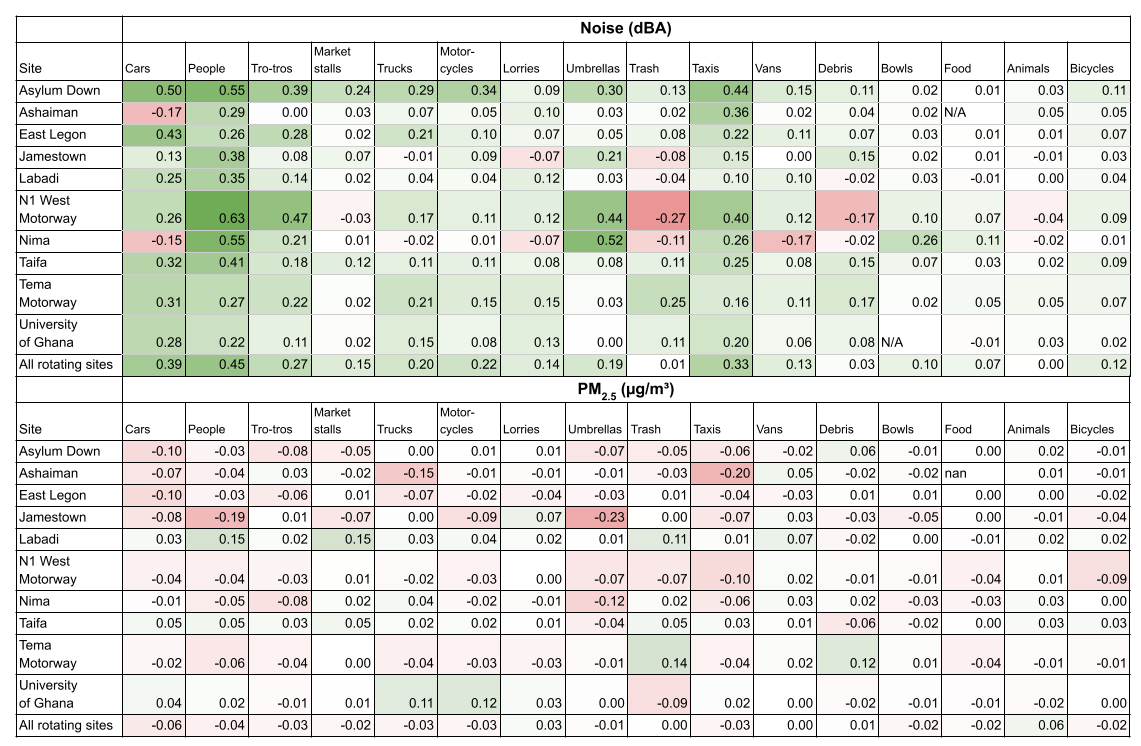


**Appendix Table E:** Spearman correlations between object counts and pollution.

For each object category, the table shows the Spearman correlation, at each fixed site, between the counts of that object as detected in images, and the corresponding pollution data. The colours from red to green indicate more negative to more positive correlations (with white centred on zero).

| **Total campaign period** | | | | | |
| --- | --- | --- | --- | --- | --- |
| Site | Red mean | Green mean | Blue mean | Gray mean | Gray SD |
| Asylum Down | 0.16 | -0.03 | -0.22 | 0.04 | -0.07 |
| Ashaiman | -0.21 | -0.29 | -0.41 | 0.32 | -0.44 |
| East Legon | 0.29 | 0.01 | -0.14 | 0.06 | -0.32 |
| Jamestown | 0 | -0.14 | -0.11 | 0.04 | -0.21 |
| Labadi | -0.1 | -0.21 | -0.3 | -0.06 | 0.07 |
| N1 West Motorway | -0.03 | -0.19 | -0.35 | -0.02 | -0.03 |
| Nima | 0.17 | 0.06 | -0.19 | 0.25 | -0.22 |
| Taifa | 0.19 | 0.06 | -0.17 | 0.07 | -0.47 |
| Tema Motorway | 0.26 | 0.02 | -0.15 | 0.27 | -0.19 |
| University of Ghana | 0.38 | 0.17 | -0.1 | 0.06 | 0.08 |

| **Harmattan period only** | | | | | |
| --- | --- | --- | --- | --- | --- |
| Site | Red mean | Green mean | Blue mean | Gray mean | Gray SD |
| Asylum Down | 0.28 | 0.04 | -0.21 | 0.24 | -0.05 |
| Ashaiman | -0.21 | -0.31 | -0.42 | 0.59 | -0.59 |
| East Legon | 0.4 | 0.19 | -0.14 | 0.13 | -0.47 |
| Jamestown | 0.15 | 0.09 | 0.33 | -0.4 | -0.74 |
| Labadi | -0.17 | -0.15 | -0.24 | -0.32 | 0.01 |
| N1 West Motorway | 0.12 | -0.01 | -0.3 | 0.12 | -0.26 |
| Nima | 0.25 | 0.16 | -0.22 | 0.39 | -0.31 |
| Taifa | 0.17 | 0.04 | -0.2 | 0 | -0.23 |
| Tema Motorway | 0.43 | 0.25 | -0.1 | 0.29 | -0.28 |
| University of Ghana | 0.44 | 0.17 | -0.05 | 0.14 | -0.12 |

| **Non-Harmattan period only** | | | | | |
| --- | --- | --- | --- | --- | --- |
| Site | Red mean | Green mean | Blue mean | Gray mean | Gray SD |
| Asylum Down | 0.16 | -0.03 | -0.22 | 0.04 | -0.07 |
| Ashaiman | -0.21 | -0.29 | -0.41 | 0.32 | -0.44 |
| East Legon | 0.29 | 0.01 | -0.14 | 0.06 | -0.32 |
| Jamestown | 0 | -0.14 | -0.11 | 0.04 | -0.21 |
| Labadi | -0.1 | -0.21 | -0.3 | -0.06 | 0.07 |
| N1 West Motorway | -0.03 | -0.19 | -0.35 | -0.02 | -0.03 |
| Nima | 0.17 | 0.06 | -0.19 | 0.25 | -0.22 |
| Taifa | 0.19 | 0.06 | -0.17 | 0.07 | -0.47 |
| Tema Motorway | 0.26 | 0.02 | -0.15 | 0.27 | -0.19 |
| University of Ghana | 0.38 | 0.17 | -0.1 | 0.06 | 0.08 |

**Appendix Table F:** Correlation of image characteristics with PM_2.5_.

For the three channels of colour in the day-time images and the overall grayscale pixels in the night-time images, the table shows the Spearman correlation between the mean, pixel intensity per image and the corresponding tagged PM_2.5_ data across all images, for each fixed site. Also included is the Spearman correlation with the standard deviation (SD) of grayscale pixel values for night-time images with PM_2.5_. The colours from red to green indicate more negative to more positive correlations, with white centred on zero.


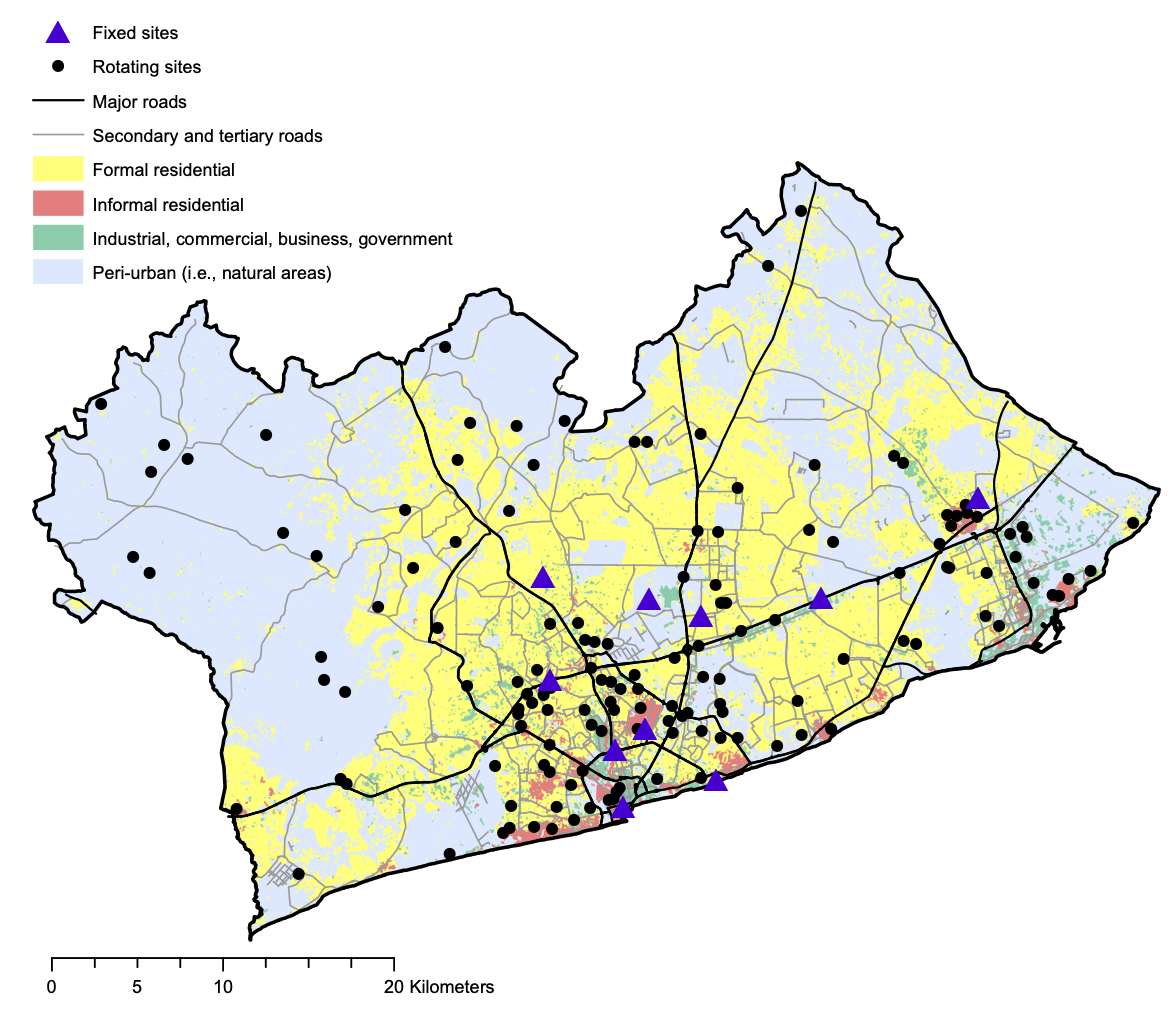


**Appendix Figure A.** Location of measurement sites.

Figure reproduced from previous work^4^ by the authors.


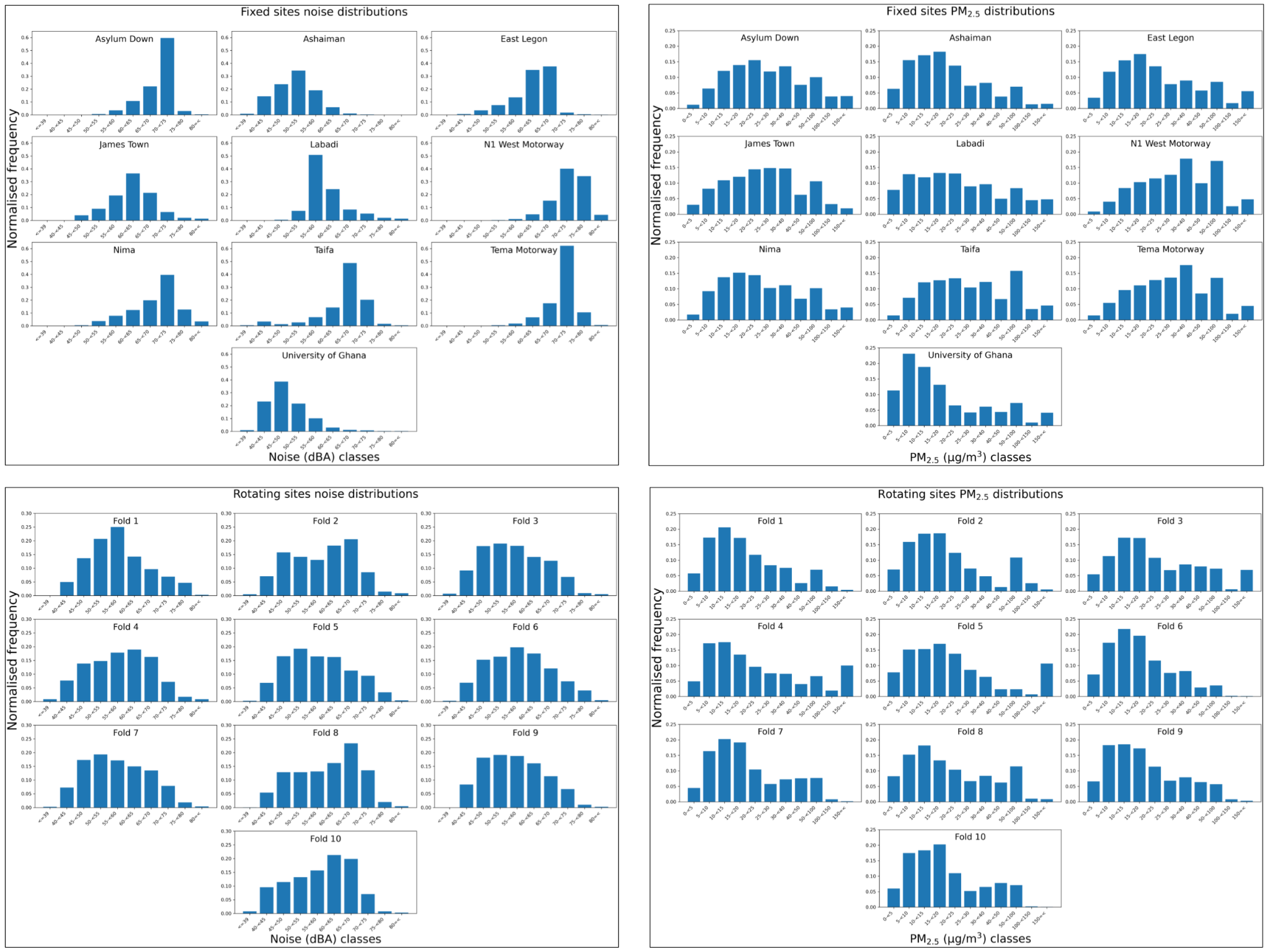


**Appendix Figure B.** Noise and PM_2.5_ distributions across sites (fixed sites) and folds (rotating sites).


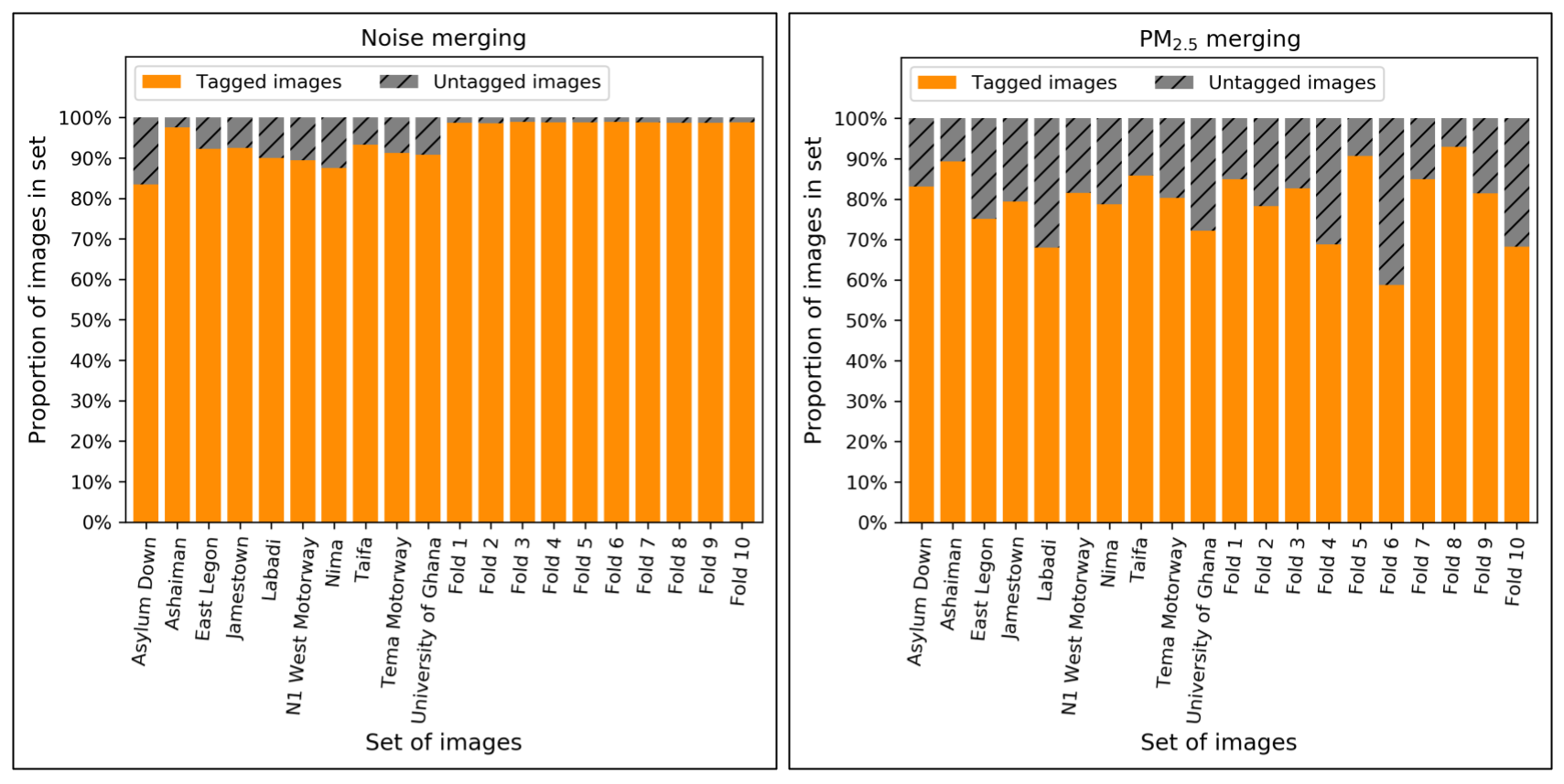


**Appendix Figure C.** Overlap of pollution and image data.

The proportion of images at each fixed site and each fold of the rotating sites tagged with noise and PM_2.5_ measurements, as described in Section 3.4 of the main paper.

**Appendix Figure D.** Objects identified in example images.

Each identified object is bounded by a box, coloured by object type. The number next to the object names shows the final layer’s activation score for the given object’s classifier, which may be heuristically interpreted as the network’s confidence score in its prediction. “Truck” refers to pick-up truck, “Bowl” refers to cooking bowl/pot, “Stall” refers to market stalls and “Stove” to cookstoves of the variety found in markets (figure reproduced from previous work^12^, by the authors).


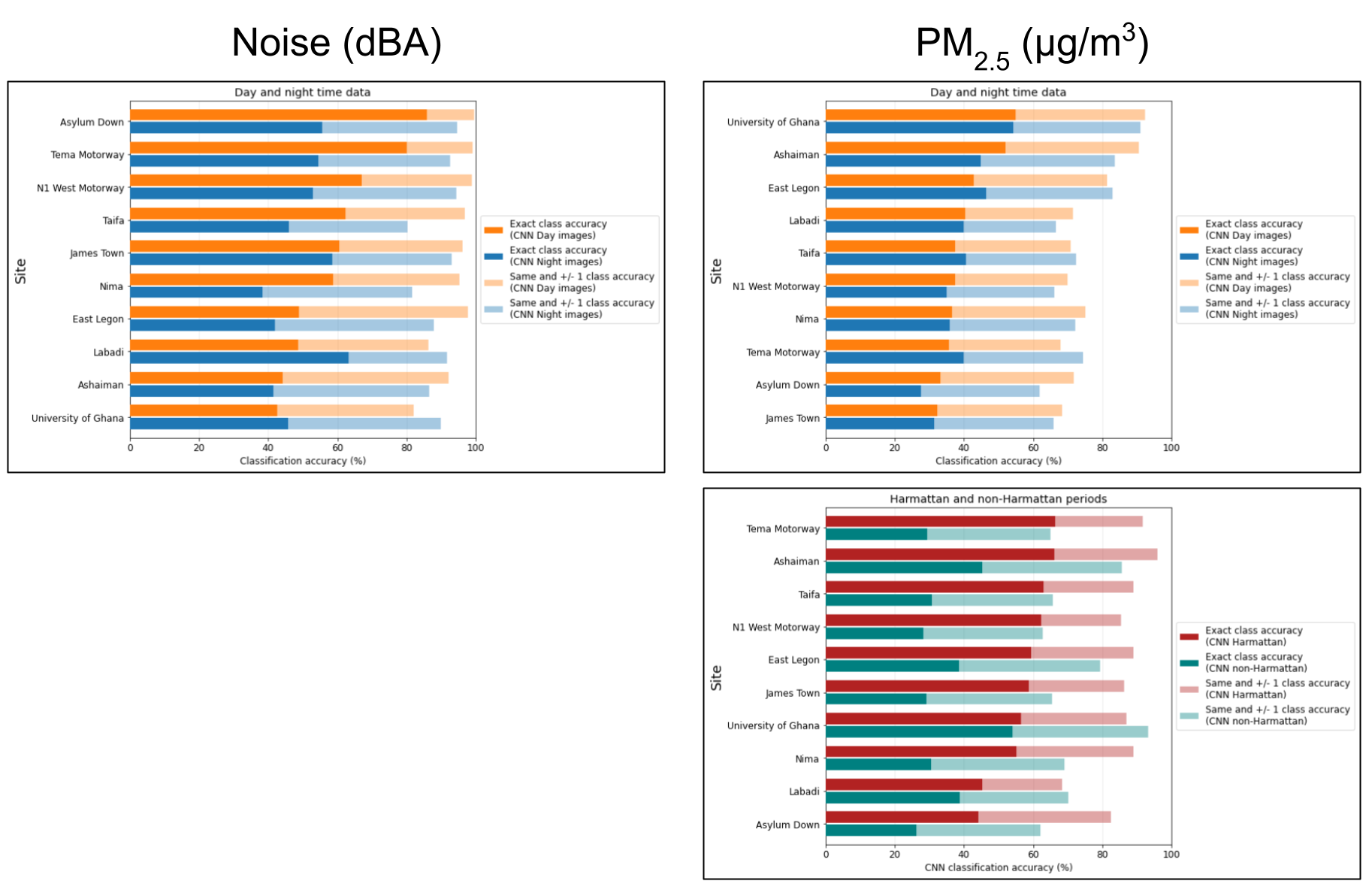


**Appendix Figure E.** Fixed site model accuracy by time of day and Harmattan season. (Question 1a)

The classification accuracy achieved by CNN and GBM models trained and tested on images from the same fixed site (Question 1a) is shown for noise and PM_2.5_, under specific environmental conditions. The top row panels show the performance of these models for day and night-time images and, and the bottom row panel for PM_2.5_ during Harmattan and non-Harmattan periods.

**Appendix Figure F.** Multiple fixed sites model accuracy (Question 2a).
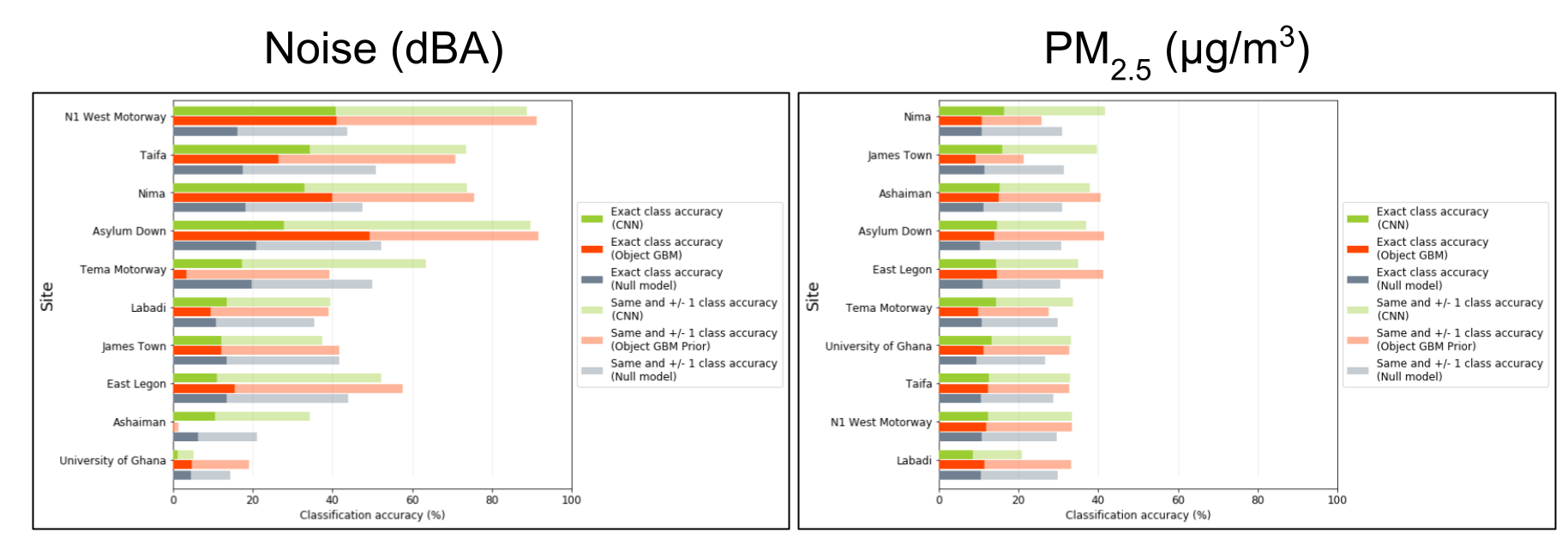


The classification accuracy achieved by CNN and GBM models trained from images at multiple fixed sites and tested on images at a different fixed site (Question 2a) for noise and PM_2.5_ prediction.

**Appendix Figure G.** Multiple rotating sites model accuracy (Question 2b).
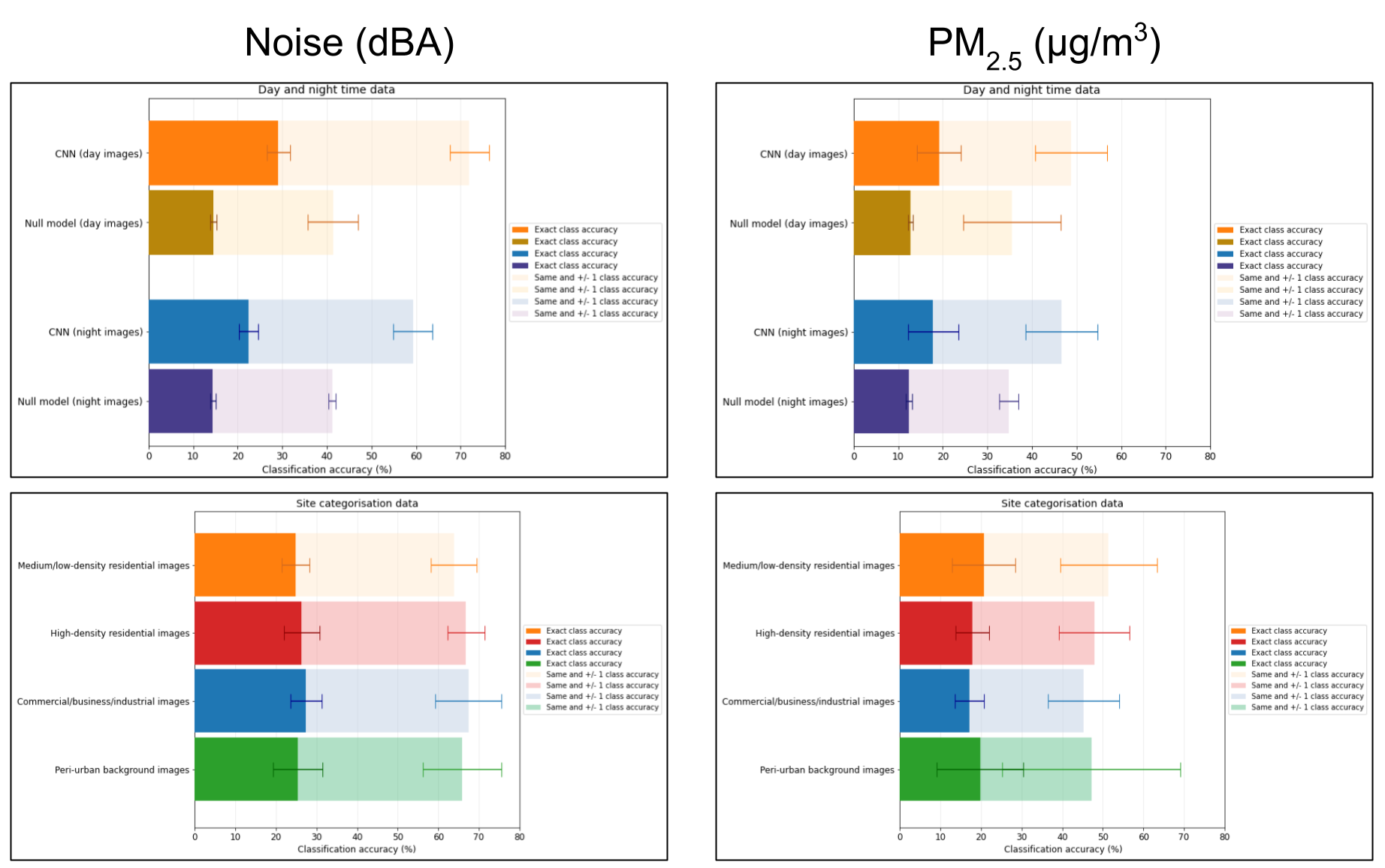


Classification accuracy achieved by CNN and GBM models trained and tested on images from rotating sites (Question 2b) for both noise and PM_2.5_ prediction, under specific environmental conditions. Accuracies are shown as the average over the folds of training data, as shown in Appendix Figure B. The bars show the standard deviation of the accuracy across different folds. The top panels show the performance of the models for day and night time images, and the bottom panels for images from different site land-use categories.

**Appendix Figure H.** Comparison of fixed vs rotating sites model accuracy.
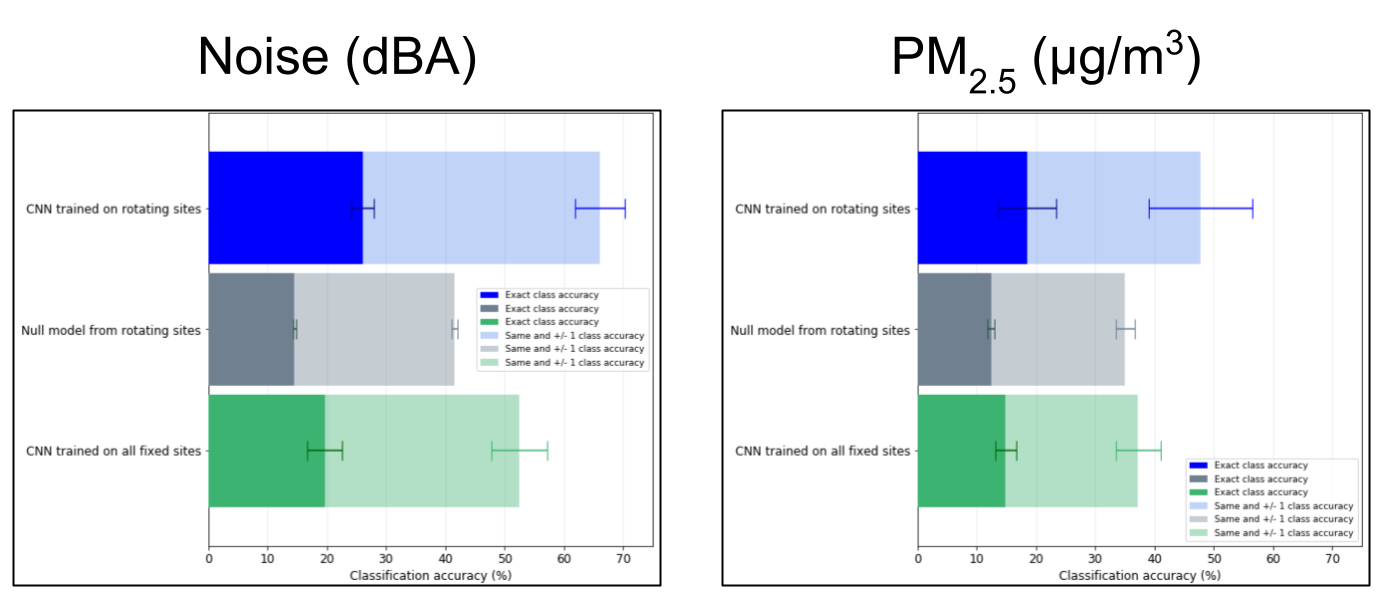


Classification accuracy achieved by CNN and GBM models trained in 2b (as in Figure 4 of the main paper) for both noise and PM_2.5_ prediction, with the addition of classification accuracy performance for models trained on data from all fixed sites, tested on the same folds of rotating sites imagery in 2c.


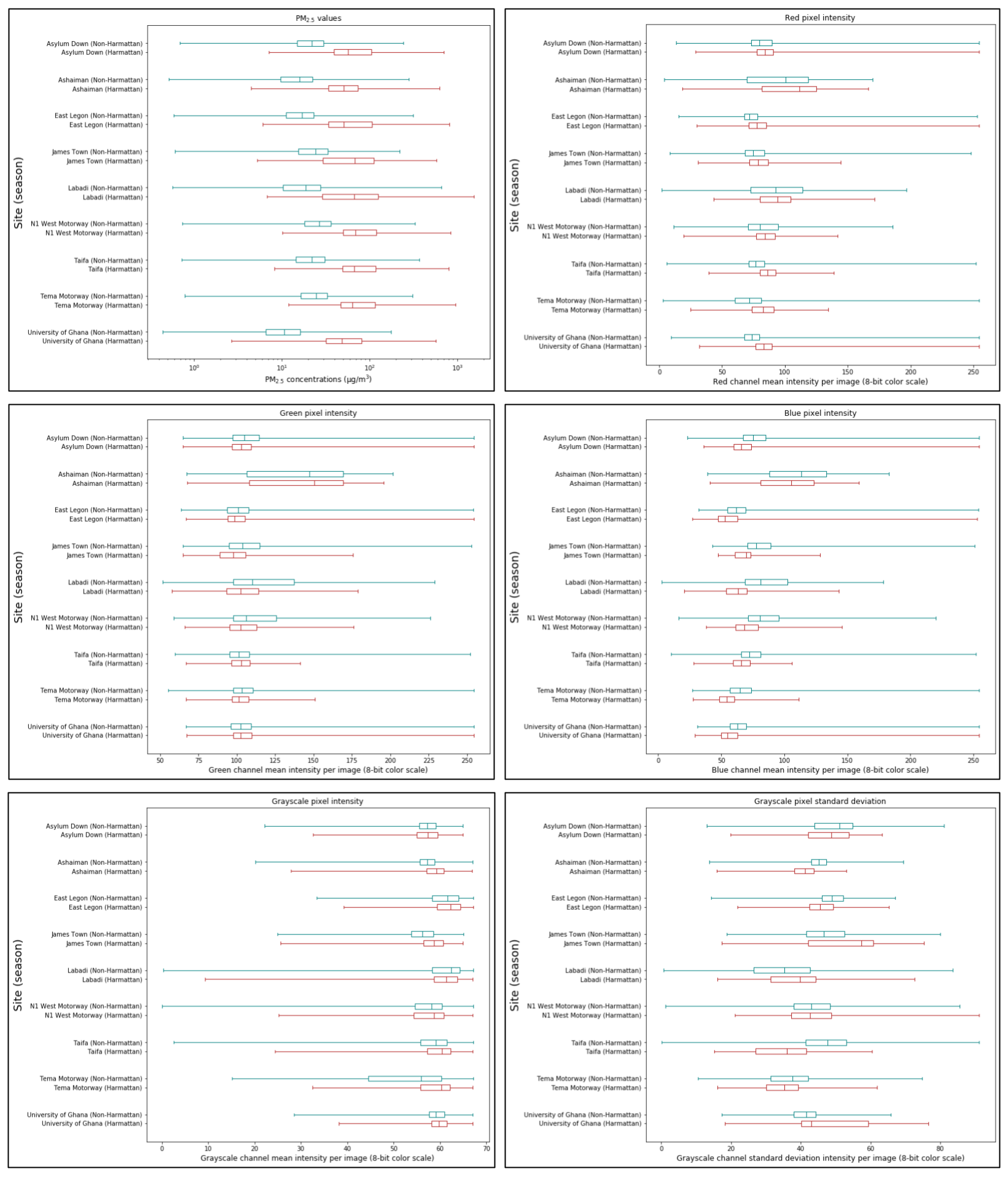


**Appendix Figure I.** Descriptive characteristics of images during Harmattan and non-Harmattan.

Each box plot shows a characteristic of the images at each fixed site, during and outside of the Harmattan season. The upper-left box plot shows the associated PM_2.5_ data for each image. The remaining box plots show mean red, green and blue pixel intensities across day images, and the mean and standard deviation of pixel intensity for night time images.

**Appendix References**

(1) ARUP and Cities Alliance. Future Proofing Cities Metropolitan Cities in Ghana. **2016**.

(2) World Bank. Rising through Cities in Ghana : Ghana Urbanization Review Overview Report, 2015. https://openknowledge.worldbank.org/handle/10986/22020.

(3) Ghana Statistical Service. Greater Accra Population. **2020**.

(4) Clark, S. N.; Alli, A. S.; Brauer, M.; Ezzati, M.; Baumgartner, J.; Toledano, M. B.; Hughes, A. F.; Nimo, J.; Bedford Moses, J.; Terkpertey, S.; Vallarino, J.; Agyei-Mensah, S.; Agyemang, E.; Nathvani, R.; Muller, E.; Bennett, J.; Wang, J.; Beddows, A.; Kelly, F.; Barratt, B.; Beevers, S.; Arku, R. E. High-Resolution Spatiotemporal Measurement of Air and Environmental Noise Pollution in Sub-Saharan African Cities: Pathways to Equitable Health Cities Study Protocol for Accra, Ghana. *BMJ Open* **2020**, *10* (8). https://doi.org/10.1136/bmjopen-2019-035798.

(5) Alli, A. S.; Clark, S. N.; Hughes, A.; Nimo, J.; Bedford-Moses, J.; Baah, S.; Wang, J.; Vallarino, J.; Agyemang, E.; Barratt, B.; Beddows, A.; Kelly, F.; Owusu, G.; Baumgartner, J.; Brauer, M.; Ezzati, M.; Agyei-Mensah, S.; Arku, R. E. Spatial-Temporal Patterns of Ambient Fine Particulate Matter (PM2.5) and Black Carbon (BC) Pollution in Accra. *Environ. Res. Lett.* **2021**, *16* (7), 074013. https://doi.org/10.1088/1748-9326/ac074a.

(6) Xie, S.; Girshick, R.; Dollar, P.; Tu, Z.; He, K. Aggregated Residual Transformations for Deep Neural Networks. In *2017 IEEE Conference on Computer Vision and Pattern Recognition (CVPR)*; IEEE: Honolulu, HI, 2017; pp 5987–5995. https://doi.org/10.1109/CVPR.2017.634.

(7) Paszke, A.; Gross, S.; Massa, F.; Lerer, A.; Bradbury, J.; Chanan, G.; Killeen, T.; Lin, Z.; Gimelshein, N.; Antiga, L.; Desmaison, A.; Kopf, A.; Yang, E.; DeVito, Z.; Raison, M.; Tejani, A.; Chilamkurthy, S.; Steiner, B.; Fang, L.; Bai, J.; Chintala, S. PyTorch: An Imperative Style, High-Performance Deep Learning Library. In *Advances in Neural Information Processing Systems*; Curran Associates, Inc., 2019; Vol. 32.

(8) Huh, M.; Agrawal, P.; Efros, A. A. What Makes ImageNet Good for Transfer Learning? *ArXiv160808614 Cs* **2016**.

(9) LeCun, Y. A.; Bottou, L.; Orr, G. B.; Müller, K.-R. Efficient BackProp. In *Neural Networks: Tricks of the Trade: Second Edition*; Montavon, G., Orr, G. B., Müller, K.-R., Eds.; Lecture Notes in Computer Science; Springer: Berlin, Heidelberg, 2012; pp 9–48. https://doi.org/10.1007/978-3-642-35289-8_3.

(10) Shorten, C.; Khoshgoftaar, T. M. A Survey on Image Data Augmentation for Deep Learning. *J. Big Data* **2019**, *6* (1), 60. https://doi.org/10.1186/s40537-019-0197-0.

(11) Belharbi, S.; Ayed, I. B.; McCaffrey, L.; Granger, E. Non-Parametric Uni-Modality Constraints for Deep Ordinal Classification. *ArXiv191110720 Cs Stat* **2020**.

(12) Nathvani, R.; Clark, S. N.; Muller, E.; Alli, A. S.; Bennett, J. E.; Nimo, J.; Bedford Moses, J.; Baah, S.; Metzler, A. B.; Brauer, M.; Suel, E.; Hughes Allison F.; Rashid, Theo; Gemmel, Emily; Moulds, Simon; Baumgartner, Jill; Toledano, Mireille; Agyemang, Ernest; Owusu, George; Agyei-Mensah, Samuel; Arku, R. E.; Ezzati, Majid. Characterisation of Urban Environment and Activity across Space and Time Using Street Images and Deep Learning in Accra. *Sci. Rep.* **2022**. https://doi.org/10.1038/s41598-022-24474-1.

(13) Friedman, J. H. Greedy Function Approximation: A Gradient Boosting Machine. *Ann. Stat.* **2001**, *29* (5), 1189–1232. https://doi.org/10.1214/aos/1013203451.

(14) Chen, T.; He, T. Xgboost: EXtreme Gradient Boosting. 4.

(15) Pedregosa, F.; Varoquaux, G.; Gramfort, A.; Michel, V.; Thirion, B.; Grisel, O.; Blondel, M.; Prettenhofer, P.; Weiss, R.; Dubourg, V.; Vanderplas, J.; Passos, A.; Cournapeau, D.; Brucher, M.; Perrot, M.; Duchesnay, É. Scikit-Learn: Machine Learning in Python. *J. Mach. Learn. Res.* **2011**, *12* (85), 2825–2830.
